# Risk factors for positive and negative COVID-19 tests: a cautious and in-depth analysis of UK biobank data

**DOI:** 10.1093/ije/dyaa134

**Published:** 2020-08-20

**Authors:** Marc Chadeau-Hyam, Barbara Bodinier, Joshua Elliott, Matthew D Whitaker, Ioanna Tzoulaki, Roel Vermeulen, Michelle Kelly-Irving, Cyrille Delpierre, Paul Elliott

**Affiliations:** d1 Department of Epidemiology and Biostatistics, School of Public Health, Imperial College London, London, UK; d2 MRC Centre for Environment and Health, Imperial College, London, UK; d3 Royal Surrey County Hospital, Guildford, Surrey, UK; d4 Department of Hygiene and Epidemiology, University of Ioannina Medical School, Ioannina, Greece; d5 Institute for Risk Assessment Sciences (IRAS), Utrecht University, Utrecht, The Netherlands; d6 UMR LEASP, Université de Toulouse III, UPS, Inserm, Toulouse, France

**Keywords:** COVID-19, SARS-CoV-2, prospective cohort, UK Biobank, infection, test data

## Abstract

**Background:**

The recent COVID-19 outbreak has generated an unprecedented public health crisis, with millions of infections and hundreds of thousands of deaths worldwide. Using hospital-based or mortality data, several COVID-19 risk factors have been identified, but these may be confounded or biased.

**Methods:**

Using SARS-CoV-2 infection test data (*n* = 4509 tests; 1325 positive) from Public Health England, linked to the UK Biobank study, we explored the contribution of demographic, social, health risk, medical and environmental factors to COVID-19 risk. We used multivariable and penalized logistic regression models for the risk of (i) being tested, (ii) testing positive/negative in the study population and, adopting a test negative design, (iii) the risk of testing positive within the tested population.

**Results:**

In the fully adjusted model, variables independently associated with the risk of being tested for COVID-19 with odds ratio >1.05 were: male sex; Black ethnicity; social disadvantage (as measured by education, housing and income); occupation (healthcare worker, retired, unemployed); ever smoker; severely obese; comorbidities; and greater exposure to particulate matter (PM) 2.5 absorbance. Of these, only male sex, non-White ethnicity and lower educational attainment, and none of the comorbidities or health risk factors, were associated with testing positive among tested individuals.

**Conclusions:**

We adopted a careful and exhaustive approach within a large population-based cohort, which enabled us to triangulate evidence linking male sex, lower educational attainment and non-White ethnicity with the risk of COVID-19. The elucidation of the joint and independent effects of these factors is a high-priority area for further research to inform on the natural history of COVID-19.

**Key Messages**

- We use data from the SARS-CoV-2 infection test (*n* = 4509, including 1325 positive and 3184 negative tests) from Public Health England, linked to the UK Biobank study (*n* = 488 083).
- Adjusting for potential confounding, male sex; Black ethnicity; social disadvantage (as measured by education, housing and income); occupation (healthcare worker, retired, unemployed); ever smoker; severely obese; comorbidities; and higher exposure to particulate matter (PM) 2.5 absorbance were independently associated with the risk of being tested for COVID-19.
- We found that male sex, non-White ethnicity, and lower educational attainment were independently associated with testing positive among tested individuals.
- None of the health risk factors or comorbidities associated with the risk of being tested were found associated with the risk of testing positive, conditional on being tested.
- We adopted complementary analytical approaches to explore the data and were able to triangulate evidence linking social factors, ethnicity and environmental exposures to COVID-19 risk.
- The elucidation of joint and independent effects of ethnicity, social and environmental factors we report is a high-priority area for further research, which may help clarify the natural history of COVID-19, and suggests possible new avenues for its prevention, diagnosis and treatment.

## Background

On 31 December 2019, in Wuhan, China, an outbreak of COVID-19 caused by the novel coronavirus SARS-CoV-2 was first reported. Since then, the infection has spread across continents and is classified as a global pandemic by the World Health Organization.^1^ As of 28 May 2020, there have been more than 5.8 million confirmed cases worldwide, and nearly 360 000 deaths; the UK is second only to the USA in number of reported COVID-19 deaths.^2^

Disease severity appears to be associated with older age, being male and having a range of comorbidities. Severe disease may result in acute respiratory distress syndrome and death.^3–10^ Disease outbreaks have led to rapid saturation of healthcare services, especially intensive care units (ICUs) in regions and conurbations in China, Europe, the USA and elsewhere.^11^^,^^12^ In response, many governments implemented quarantine measures^13^ to curtail the spread of infection and limit the number of avoidable deaths.

In the UK, COVID-19 was first documented at the end of January, 2020, although regression-based modelling has inferred probable community spread before detection of first cases in many Western countries.^14^ Early in the epidemic, testing included community cases with typical symptoms or people returning from high-risk areas; this approach was abandoned and testing was then almost exclusively reserved for patients presenting to hospital with high suspicion of COVID-19 based on symptoms and/or clinical/radiological findings.^15^ By 23 March, 6650 cases had been reported in the UK and a nationwide lockdown was implemented.

We present here an analysis of UK Biobank data identifying risk factors for testing positive or negative for SARS-CoV-2 infection up to 18 May 2020, as well as those discriminating test positive vs test negative individuals using a test negative design approach.^16^

## Study and methods

### Study population

UK Biobank is a population-based prospective cohort including 502 506 volunteers with active consent, aged 40–69 years at recruitment from 2006 to 2010. At the latest follow-up, 14 423 participants had died leaving *n* = 488 083 participants for the present analyses.^17^ Each participant provided data on lifestyle, exposures, sociodemographic factors, medical history and medications.

Results of COVID-19 tests from Public Health England’s Second Generation Surveillance System microbiology database were linked to UK Biobank participants.^18^ These only included ‘Pillar I’ data, i.e. swab testing in Public Health England (PHE) labs and NHS hospitals for those with a clinical need, and health and care workers. Samples were analysed using a reverse transcriptase-polymerase chain reaction (RT-PCR) test for SARS-CoV-2. Most of the samples are from combined nose/throat swabs (67%) and upper respiratory tract (25%). In intensive care settings, lower respiratory samples may also be analysed. These included results of 7539 tests from 4509 UK Biobank participants (1824 tested more than once) between 16 March and 18 May 2020 (as available 25 May 2020). Tested participants were classified positive if at least one of their test results was positive, and negative otherwise. Tested participants were considered inpatients if reported as such in the microbiological record for at least one of their tests (*n* = 3186). Inpatient tests arise from specimens collected from an acute (emergency) care provider, an A&E department or an inpatient location. However, microbiology data source has not been linked to admissions data. It can therefore happen that data reported as being from outpatients can arise from an inpatient.

### Participant characteristics

Variables were grouped into five categories: demographic, social, health risk, medical factors and environmental exposures (Supplementary Table 1A, available as Supplementary data at *IJE* online).

Demographic variables comprised age calculated as of 31 January 2020, at the time of the first diagnosed UK COVID-19 case, sex and ethnicity, defined as White, Black and Other (South Asian or other ethnic groups).

Social variables included education measured by highest level of qualification attained in three categories: high (College or University degree), intermediate (A/AS levels, O levels/GCSEs, CSEs, NVQ or HND, or equivalent, and other professional qualifications) and low (none of the above). Housing was (i) type of accommodation (house/bungalow or flat), (ii) whether rented or owned, and if owned outright or with a mortgage, and (iii) number of individuals in household. Average household income was included in four categories: <GBP 18 000; GBP 18 000–30 999; GBP 31 000–51 999; >GBP 52 000. Occupation at recruitment was categorized as: retired, employed healthcare workers (including health professionals, health and social welfare associated professionals, healthcare and related personal services, health and social service managers and hospital porters), employed non-healthcare workers (in paid employment or self-employed) and unemployed (including studying and doing unpaid voluntary work).

Health risk factors were smoking, alcohol drinking (current, former or never), and body mass index (BMI) categorised as: <25, 25–30, 30–40 and >40 kg/m^2^. Comorbidities were derived from self-reported illness at baseline and diagnoses from linked Hospital Episode Statistics data (yes/no): (i) cancer, (ii) cardiovascular disease, (iii) hypertension, (iv) diabetes, (v) respiratory disease, and (vi) autoimmune disease (Supplementary Table 1B, available as Supplementary data at *IJE* online). Number of reported medications at recruitment was categorized as: 0, 1, >1.

Finally, modelled levels of environmental exposure (continuous variables) to nitrogen oxides (NO_*x*_) and particulate matter for particle of diameter smaller that 10 or 2.5 micrometers (PM10, PM2.5), and to soot (PM2.5 absorbance), were estimated from residential address in 2010 using land-use regression models at the European level.^19^

### Statistical analyses

**Figure 1:**
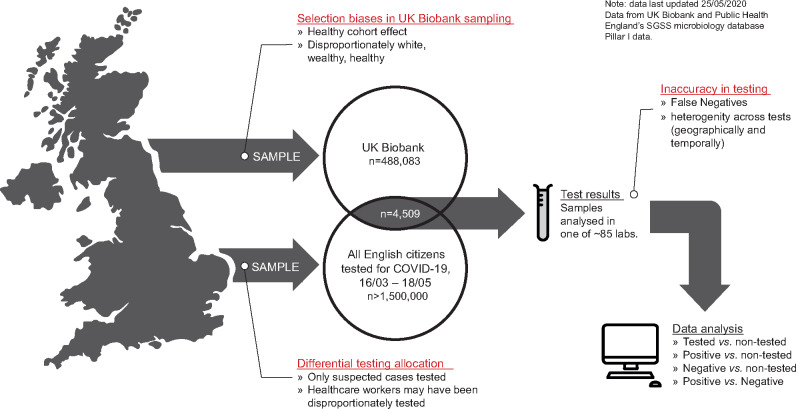
Overview of the data workflow, depicting the synthesis of data from the UK Biobank for COVID-19 testing data. Key biases that are innate to the data gathering processes and test allocation are annotated, as these impact the statistical inferences that can be made from the data.

We compared means or proportions for each covariate between tested and non-tested participants (Figure 1); differences between the two populations were assessed using Student’s *t*-test (continuous variables: age and exposure levels) and chi-squared test (categorical variables). We compared (i) the tested and non-tested populations at two stages of the UK epidemic (before and after 10 April 2020), (ii) inpatients and outpatients, and (iii) healthcare and non-healthcare workers.

We used univariate logistic regression to model for each covariate the risk of (i) being tested for COVID-19 (tested vs non-tested), (ii) test positive (confirmed case) vs non-tested, (iii) test negative (suspected case) vs non-tested. In order to account for a potential bias in the decision to test and heterogeneity among the participants who tested negative, some of whom may have had illnesses other than COVID-19, we adopted test-negative case-control design,^16^ modelling the risk of testing positive conditional on being tested. Such design circumvents some of the selection biases as the analyses are restricted to symptomatic people that were tested. To implement this approach we modelled the risk of testing positive in the tested population only. Continuous covariates (age and environmental exposures) were standardized to ensure comparability and resulting odds ratios (ORs) were expressed as the risk change for a one standard deviation increase in the value of the covariate.

For the four aforementioned analyses, we additionally accounted for correlation across covariates using logistic Least Absolute Shrinkage and Selection Operator (LASSO) regression to model joint effects,^20^ calibrated using 10-fold cross-validation minimizing the binomial deviance. We investigated stability of the variables selected by fitting logistic LASSO models on (*n* = 1000) random 80% sub-samples of the full population. As a measure of relevance for each variable, we report its selection proportion.^21^

To account for multiple confounding, we sequentially adjusted for (i) age and sex (base model), (ii) social factors, (iii) health risk factors, (iv) medical variables (comorbidities and number of medications), and (v) environmental factors.

Sensitivity analysis was conducted by separately considering models with healthcare workers and non-healthcare workers.

All analyses were performed in R, version 3.6.3.

## Results

### Descriptive statistics and univariate analyses

**Figure 2:**
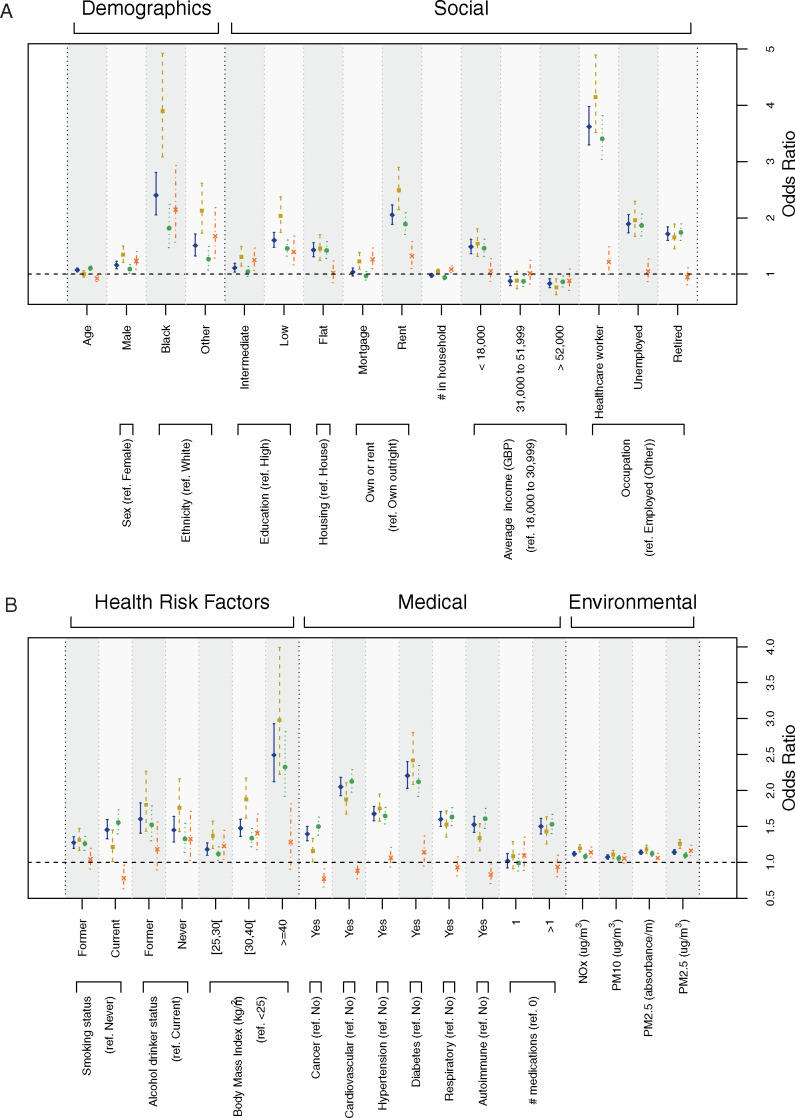
Results from the univariate logistic models predicting from each covariate separately the risk of (i) being tested for COVID-19 (outcome: tested vs non-tested, in blue, plain line), (ii) being tested positive for COVID-19 (outcome: tested positive vs non-tested, in beige dashed line), (iii) being tested negative for COVID-19 (outcome: tested negative vs non-tested, in green, dotted line), and (iv) being tested positive conditional on being tested (outcome: tested positive vs tested negative, in orange dashed/dotted line). Effect size estimates are expressed as odds ratios and are represented for demographic covariates and social factors (**A**), health risk, medical and environmental factors (**B**).

Descriptive statistics comparing the UK Biobank non-tested population (*n* = 483 574) and those tested for COVID-19 (*n* = 4509 tested individuals, *n* = 1325 positive, and *n* = 3184 negative) are shown in Table 1. The distribution of tested participants in relation to the number of tests they underwent is summarized in Supplementary Table 2, available as Supplementary data at *IJE* online, and shows an excess of men in those who were tested more than twice. Results of univariate logistic models are shown in Figure 2 and Supplementary Table 3, available as Supplementary data at *IJE* online.

**Table 1:** Characteristics of the non-tested and tested populations for COVID-19 in the UK Biobank study. For each variable, the difference between the tested and non-tested populations is evaluated using a Student’s *t*-test (for age and environmental exposures) comparing the mean values in the tested and non-tested populations or a chi-squared test (for categorical variables)

		Non-tested (n = 483 574)		Tested (n = 4509)		P-value (tested vs non-tested)
		n	Mean (SD)/proportion	n	Mean (SD)/proportion	
Demographics	Age (years)	483 574	68.06 (8.10)	4509	68.64 (8.88)	1.66 × 10^−5^
	Sex					7.35 × 10^−7^
	Female	265 408	54.88%	2308	51.19%	
	Male	218 166	45.12%	2201	48.81%	
	Ethnicity					9.42 × 10^−36^
	White	454 766	94.56%	4067	90.74%	
	Black	7779	1.62%	167	3.73%	
	Other	18 385	3.82%	248	5.53%	
**Social**	Education					8.39 × 10^−31^
	High	156 615	33.04%	1250	28.53%	
	Intermediate	237 498	50.11%	2108	48.12%	
	Low	79 887	16.85%	1023	23.35%	
	Type of accommodation					4.94 × 10^−16^
	House	431 992	90.13%	3826	86.46%	
	Flat	47 286	9.87%	599	13.54%	
	Own or rent accommodation					6.71 × 10^−70^
	Own outright	248 573	52.65%	2057	47.27%	
	Own with a mortgage	178 749	37.86%	1535	35.27%	
	Rent	44 782	9.49%	760	17.46%	
	Number in household	479 386	2.44 (1.32)	4421	2.42 (1.52)	2.29 × 10^−1^
	Average household income (GBP)					1.25 × 10^−46^
	<18 000	91 289	22.27%	1197	32.02%	
	18 000–30 999	103 946	25.35%	918	24.56%	
	31 000–51 999	107 804	26.29%	834	22.31%	
	>52 000	106 960	26.09%	789	21.11%	
	Occupation					1.19 × 10^−179^
	Unemployed	66 382	13.89%	784	17.61%	
	Employed (healthcare worker)	27 789	5.81%	628	14.11%	
	Employed (other)	239 832	50.17%	1497	33.63%	
	Retired	144 030	30.13%	1542	34.64%	
**Health risk factors**	Smoking status					8.89 × 10^−21^
	Never	266 023	55.33%	2169	48.49%	
	Previous	165 298	34.38%	1718	38.41%	
	Current	49 474	10.29%	586	13.10%	
	Alcohol drinker status					1.75 × 10^−18^
	Never	21 434	4.45%	278	6.20%	
	Previous	16 816	3.49%	241	5.37%	
	Current	443 765	92.06%	3968	88.43%	
	Body mass index (kg/m^2^)					7.93 × 10^−40^
	<25	159 707	33.22%	1221	27.42%	
	25–30	204 524	42.54%	1847	41.48%	
	30–40	107 471	22.35%	1212	27.22%	
	≥40	9076	1.89%	173	3.89%	
**Medical**	Cancer					1.19 × 10^−19^
	No	405 967	83.95%	3560	78.95%	
	Yes	77 607	16.05%	949	21.05%	
	Cardiovascular					2.05 × 10^−118^
	No	387 208	80.07%	2985	66.20%	
	Yes	96,366	19.93%	1524	33.80%	
	Hypertension					3.00 × 10^−67^
	No	326 106	67.44%	2492	55.27%	
	Yes	157 468	32.56%	2017	44.73%	
	Diabetes					4.78 × 10^−79^
	No	449 911	93.04%	3870	85.83%	
	Yes	33 663	6.96%	639	14.17%	
	Respiratory					2.12 × 10^−47^
	No	381 403	78.87%	3157	70.02%	
	Yes	102 171	21.13%	1352	29.98%	
	Autoimmune					9.22 × 10^−30^
	No	415 457	85.91%	3607	80.00%	
	Yes	68 117	14.09%	902	20.00%	
	Number of medications					4.43 × 10^−37^
	0	134 487	27.86%	988	21.96%	
	1	92 200	19.10%	690	15.33%	
	>1	256 082	53.04%	2822	62.71%	
**Environmental**	NO_*X*_ (µg/m^3^)	476 556	44.06 (15.50)	4442	46.06 (15.93)	7.76 × 10^−17^
	PM10 (µg/m^3^)	443 941	16.24 (1.90)	4438	16.37 (1.85)	1.16 × 10^−6^
	PM2.5 (absorbance/m)	443 941	1.19 (0.27)	4438	1.23 (0.29)	1.09 × 10^−19^
	PM2.5 (µg/m^3^)	443 941	9.99 (1.06)	4438	10.14 (1.08)	9.34 × 10^−20^

The probability of being tested was significantly higher in older individuals, among men and people of non-White ethnicity. Tested individuals were more likely to be of lower socio-economic status (SES): having lower educational attainment, living in (i) a flat, (ii) rented accommodation, (iii) a household with an average income <GBP 18 000/year and less likely to be from a household with an average income >GBP 31 000/year. In addition, tested individuals were more likely to have been retired, healthcare workers, unemployed, ever smokers, former or never drinkers, overweight, obese and severely obese. Comorbidities were associated with an increased risk of being tested: cancer, cardiovascular disease, hypertension, diabetes, respiratory disease, autoimmune disease and reporting use of more than one medication. Tested participants were also exposed to higher levels of air pollutants at residence (Figure 2 and Supplementary Table 3, available as Supplementary data at *IJE* online).

Most of these associations (direction and statistical significance) also held for test positive or test negative separately compared to the non-tested population (Figure 2, Supplementary Table 3, available as Supplementary data at *IJE* online).

Among the tested population, the risk of testing positive compared with testing negative was (i) higher in men, non-White individuals, participants with lower educational attainment, renting and owning with a mortgage, living with more people, never drinkers, overweight and obese individuals, and those exposed at residence to higher environmental levels of NO_*x*_ and PM2.5, and (ii) slightly lower in older individuals, current smokers, participants previously diagnosed with cancer or autoimmune disease (Supplementary Table 3, available as Supplementary data at *IJE* online).

Comparison of the tested populations in the first and second parts of the UK epidemic (before and from 10 April 2020) showed some differences in that from 10 April, tested participants were younger, included fewer men, had slightly higher SES and fewer comorbidities (Supplementary Table 4, available as Supplementary data at *IJE* online). Compared with outpatients, inpatients were more likely to be older, White, of male sex, of lower SES, to have underlying comorbidities and to be on more than one medication (Supplementary Table 5, available as Supplementary data at *IJE* online).

The healthcare workers tested for COVID-19 differed from the rest of the tested population according to all characteristics except environmental exposures and type of accommodation. In particular, they were younger, more affluent (as measured by income), showed a lower prevalence of all comorbidities, a higher proportion of non-White and lean individuals, of women and of never-smokers (Supplementary Table 6, available as Supplementary data at *IJE* online).

### Multivariate and attenuation analyses

**Figure 3:**
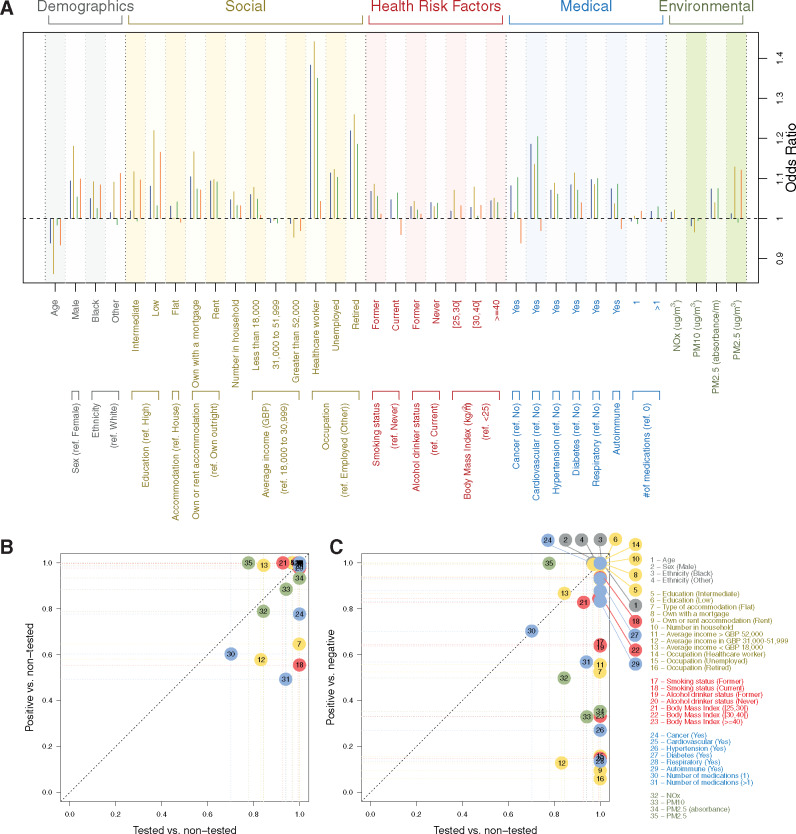
Penalized odds ratios from logistic-LASSO models regressing jointly all predictors against the risk of (i) being tested for COVID-19 (outcome: tested vs non-tested, in blue), (ii) being tested positive for COVID-19 (outcome: tested positive vs non-tested, in beige), (iii) being tested negative for COVID-19 (outcome: tested negative vs non-tested, in green), and (iv) being tested positive conditionally on being tested (outcome: tested positive vs tested negative, in orange) (**A**). Selection proportion from stability analysis of the LASSO for (i) the risk of testing positive in the full population (**B**), and (ii) the risk of testing positive for COVID-19 conditionally on being tested (*y*-axis) (**C**) against the selection proportion for the model of the probability of being tested (*x*-axis). Selection proportions were inferred from 1000 models based on an 80% subsample of the population and are reported for each of the demographics (in grey, *n* = 4), social (brown, *n* = 12), health risk (red, *n* = 7), medical (blue, *n* = 8), and environmental (green, *n* = 4) factors.

We used logistic LASSO models to account for correlation and joint contribution across covariates. Being younger, a man, of non-White background, ever smoker, non-drinker, overweight, obese or severely obese, on more than one medication, of lower SES, exposed to higher environmental levels of NO_*x*_, PM2.5 or PM2.5 absorbance or having comorbidities were all found to jointly contribute to a higher probability of being tested (Figure 3a, blue). Models for the probability of being a confirmed or suspected case in the full UK Biobank population (Figure 3a, beige and green, respectively) selected fewer but consistent sets of predictors (*n* = 33 and 32 respectively). Models for the risk of testing positive, conditional on being tested (Figure 3A, orange), selected 26 covariates notably including age, gender, ethnicity, educational attainment and occupation, obesity, having had a cancer, diabetes, cardiovascular or autoimmune disease, using one or more medication and environmental exposures.

Stability analyses showed that the frequently selected variables to predict being a confirmed case (Figure 3B, *n* = 28 variables with selection proportion >80%) were also selected to predict the probability of being tested (irrespective of the outcome of the test), with a selection proportion close to 100% (except for PM2.5 and household income >GBP 52 000 whose selection proportions were 78 and 85% respectively). Eighteen variables jointly differentiated testing positive vs testing negative with selection proportion >80%: being younger, a man, of non-White ethnicity, of lower educational attainment, owning with a mortgage, living with more people, lower income, being a healthcare worker, currently smoking, being overweight or obese, exposed to higher levels of PM2.5, previously diagnosed with cancer, diabetes, cardiovascular or autoimmune disease.

**Figure 4:**
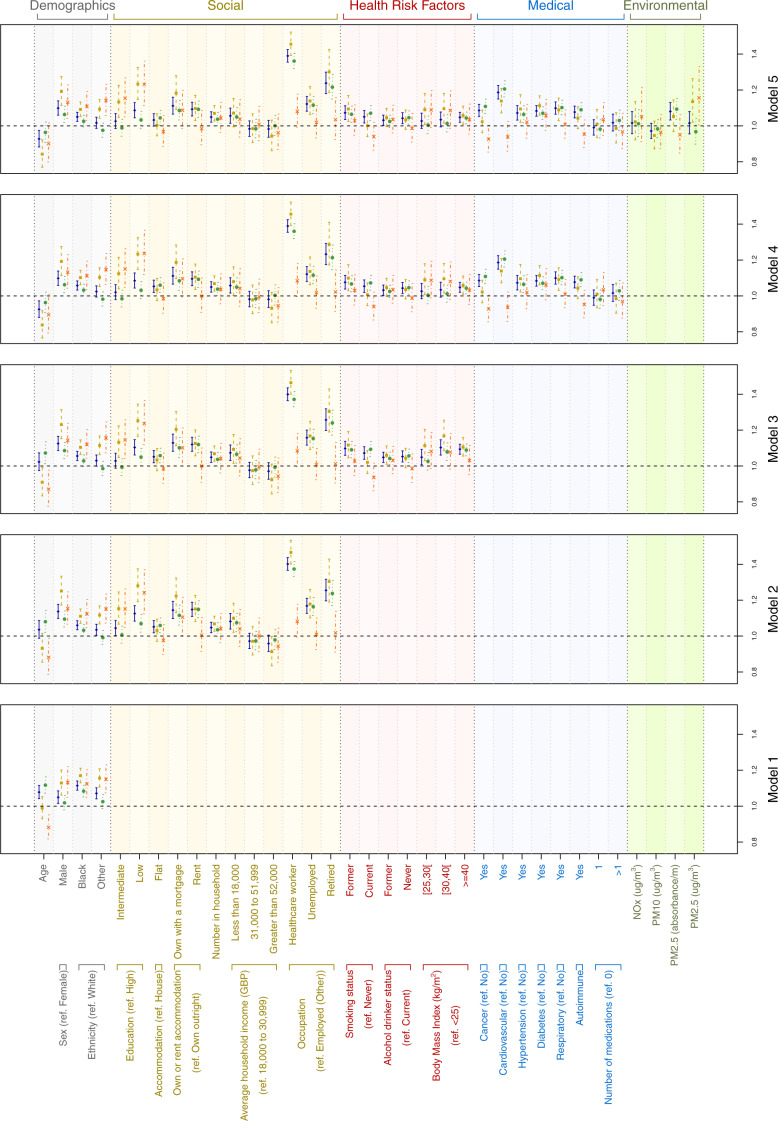
Odds ratio (95% confidence intervals), from the logistic models for the probability of (i) being tested for COVID-19 (blue, plain line), (ii) testing positive (beige, dashed line), (iii) testing negative (green, dotted line), and (iv) testing positive conditionally on being tested (orange, dashed/dotted line). Results are represented for a sequentially adjusted model, where benchmark predictors are defined as demographic descriptors (Model 1), and models are additionally adjusted for social (Model 2), health risk (Model 3), medical (Model 4) and environmental factors (Model 5).

In the fully adjusted model (Figure 4), variables independently associated with testing positive or negative with odds ratio (OR) ≥ 1.05 were (Supplementary Table 7A, available as Supplementary data at *IJE* online): male sex (OR = 1.10 [1.06–1.14], *P* = 3.43 × 10^−7^); Black vs White ethnicity (OR = 1.05 [1.02–1.08], *P* = 7.55 × 10^−5^); low vs high educational attainment (OR = 1.09 [1.05–1.13], *P* = 2.84 × 10^−5^); owning a house with a mortgage or renting vs owning outright (OR > 1.09, *P* < 10^–6^); number in household (OR = 1.05 [1.02–1.08], *P* = 6.17 × 10^−4^); earning <GBP 18 000 per year (OR = 1.06 [1.01–1.10], *P* = 8.16 × 10^−3^); being a healthcare worker, unemployed or retired vs other employed (OR > 1.12, *P* < 10^–9^); current or former smoker vs never smoker (OR > 1.05, *P* < 10^–2^); severely obese vs non-overweight (OR = 1.05 [1.02–1.08], *P* = 5.72 × 10^−4^); having had cancer, cardiovascular disease, hypertension, diabetes, respiratory disease, autoimmune disease (OR > 1.07, *P* < 10^–3^ for all comorbidities). The probability of being tested was inversely associated with age (OR = 0.93 [0.88–0.97], *P* = 2.81 × 10^-3^).

The strength of the associations between the probability of being tested and the levels of environmental exposure to NO_*x*_, PM10, PM2.5 were attenuated, and only PM2.5 absorbance was associated in the fully adjusted model (OR = 1.08 [1.03–1.13], *P* = 7.99 × 10^−4,^Supplementary Table 7A, available as Supplementary data at *IJE* online).

The fully adjusted models restricted to confirmed or suspected cases (Figure 4, beige and green) gave broadly similar results, except that being younger, of non-White ethnicity, of low educational attainment, a former drinker, which were not associated with risk in suspected cases; and having an average household income <GBP 18 000, being a current smoker, a non-drinker, previously diagnosed with cancer or autoimmune disease, or exposed to higher levels of PM2.5 absorbance were not associated with risk in confirmed cases; and being overweight or obese (OR > 1.09, *P* < 0.03), which were associated with risk in confirmed cases (Figure 4).

Of the associations identified in the univariate analyses for risk of testing positive in the tested population, male sex (OR = 1.13 [1.04–1.23], *P* = 3.20 × 10^−3^), lower educational attainment (OR > 1.15, *P* < 10^–2^), non-White ethnicity (OR > 1.11, *P* < 10^–2^), and PM2.5 (OR = 1.16 [1.00–1.33], *P* = 4.24 × 10^−2^) were weakly associated with test positive vs test negative in the fully adjusted model (Figure 4, Supplementary Table 7B, available as Supplementary data at *IJE* online).

### Sensitivity analysis

Excluding healthcare workers from our analyses did not materially affect our results (Supplementary Figure 1A and B, available as Supplementary data at *IJE* online). Results from models restricted to healthcare workers were similar to those from non-healthcare workers (Supplementary Figure 1C and D, available as Supplementary data at *IJE* online).

## Discussion

### Main findings

We found important differences between those tested for SARS-CoV-2 infection and the rest of the UK Biobank cohort. Accounting for potential confounding, we found, in a fully adjusted model, that male sex; Black ethnicity; social disadvantage (as measured by education, housing and income); being a healthcare worker, unemployed or retired; a current or former smoker; severely obese; comorbidities (cancer, cardiovascular disease, respiratory or autoimmune diseases); and greater exposure to PM2.5 absorbance were all independently associated with the risk of being tested for COVID19. We found that the associations linking obesity and the risk of being tested were strongly attenuated while adjusting for comorbidities. We found consistent results when comparing only confirmed COVID-19 cases with the non-tested population. Additionally, comparing data for test positive and test negative individuals within the tested population, we found, in a fully adjusted model, consistent associations linking the risk of testing positive and male sex, lower educational attainment, non-White ethnicity and PM2.5.

Health risk factors and comorbidities were found to be associated with the risk of being tested but not with the risk of testing positive, conditional on being tested in a fully adjusted model. This suggests that these factors may help in predicting the risk of developing COVID-19 symptoms, and therefore the probability of receiving a test. Nevertheless, within the tested population, these factors do not provide any further information that would be relevant to predicting the outcome of the test.

Given the high specificity of the RT-PCR test for SARS-CoV-2, it is likely that all test positive individuals were true cases. Some who tested negative may have had other illnesses with similar clinical presentation and possibly shared risk factors. This is in keeping with the limited and variable sensitivity of the RT-PCR test for SARS-CoV-2 (reportedly ∼70%)^22^ and the possibility that those testing negative may have presented to healthcare at a point in the disease course when SARS-CoV-2 RNA may no longer be detectable in the sampled tissues.^23^

Nonetheless and despite these limitations we were able to find consistent evidence linking social factors, ethnicity, and marginally, higher levels of environmental exposures, to COVID-19 risk.

Our results add to those from ICU data,^24^ which showed greater risk of ICU admission for men and individuals from a non-White background. This may explain that we observed an excess of men, who appear to be at higher risk of a severe form of COVID-19, in those who were tested more than twice for the infection. Additionally, severe COVID-19 cases in the ISARIC consortium data^25^ were more likely to be men and have comorbidities such as cardiovascular or respiratory disease. Furthermore, data from the UK Office for National Statistics show that non-White individuals (specifically from Black, Bangladeshi and Pakistani backgrounds) are 1.6–1.9 times more likely to die from COVID-19 after adjustment for SES.^26^ Other studies further corroborate the increased risk associated with being male,^27^ diabetic,^27^ non-White^27–29^ and of a lower SES.^27^^,^^30^^,^^31^ Whereas the ICU data included a comparator group with non-COVID-19 viral pneumonia, ISARIC data include COVID-19 cases only. In the present work the cases were drawn from the extensively evaluated UK Biobank cohort; this made it possible to directly compare the characteristics of confirmed or suspected COVID-19 cases with the non-tested population and to evaluate the mutually independent effects of multiple variables on risk.

It has been hypothesized that the higher rate of severe COVID-19 among non-White individuals may reflect lower SES and higher prevalence of comorbidities in Black and minority ethnic groups.^32^ In the present study, we show that each of these factors independently contributes to the risk of being a confirmed or suspected COVID-19 case. Results from our test negative design approach suggest that non-White ethnicity is associated with increased risk of COVID-19, independently from social factors and comorbidities. Although it is possible that our results may reflect some residual confounding by SES and/or comorbidities, our results are suggestive that non-White individuals have increased risk of severe illness and death from COVID-19. If so, the reasons for these increased risks are unknown and indicate an area for urgent future research.

There has been speculation regarding the extent to which healthcare workers may be at increased risk of COVID-19.^33^ Although it is possible that healthcare workers might have been preferentially tested, this was not widespread policy at the time of study,^34^ and our results did not materially change with or without inclusion of healthcare workers. It has been suggested that the higher risk in healthcare workers may reflect higher or repeated exposure to SARS-CoV-2 and to aerosol-generating procedures^35^^,^^36^ as well as reported lack of adequate personal protective equipment at that time.^35–38^

Comorbidities are also associated with lower SES, but again we found that they were independently and jointly contributing to increased risk, specifically, cancer, cardiovascular disease, hypertension, diabetes, respiratory and autoimmune diseases. Unlike other studies reporting higher risk in obese individuals,^39^^,^^40^ and postulating ACE2 expression in adipose tissue as a potential mechanism for the role of obesity in severe COVID-19,^41^ we found that the association of obesity and COVID-19 was attenuated while adjusting for comorbidities. These included cardiovascular diseases, diabetes and respiratory disease, which were the most strongly associated with the risk of developing COVID-19 symptoms, independently of obesity. This points to a potential role for metabolic and pro-inflammatory disorders in the development of COVID-19. Furthermore, we found an excess of ever smokers in the tested group, in agreement with some studies^6^ but not others^27^—although the former may have been affected by collider bias.^42^

Although increased risks from higher levels of outdoor air pollutants at the person’s residence was seen in the unadjusted analysis, these were attenuated after multiple adjustment and only associations involving PM2.5 were (borderline) statistically significant. This suggests that previous reports of an association between outdoor air pollution and COVID-19^40^^,^^43^ may have been confounded. Nonetheless, given the effects of outdoor air pollution on respiratory function,^44^ cardiovascular disease^45^ and other infections such as SARS,^46^ this area requires further investigation.

Although age is understood to be a major risk factor for severe disease or death from COVID-19,^47^ age was found to be inversely associated with the risk of being tested but not with the risk of testing positive among the tested population. However, being retired, which remained significant in our adjusted models, is a proxy for older age, and may explain the inverse association with age in the multiply adjusted models. Additionally, older people might have been less likely to have been tested, either due to testing protocols or concerns about attending healthcare settings due to shielding,^48^ and non-referral to hospital, e.g. from care homes where residents were not routinely tested.

## Limitations

First, UK Biobank is not representative of the general UK population due to healthy volunteer selection bias and over-representation of White people, participants of higher SES and certain occupations.^49^ In particular, our study population included higher numbers of healthcare workers than in the general population.^50^ This could further be explained by the fact that we adopted a broader definition of healthcare workers (including health and social service manager and care assistants and home carers). Nonetheless, the range of factors influencing the risk of confirmed or suspected COVID-19 concurs with and extends findings from other studies of hospital-only populations and national mortality data. We also show that our results and conclusions are robust to the exclusion of healthcare workers, which indicates that despite the non-representativeness of the UK Biobank population, our results are not biased by the over-representation of healthcare workers.

Second, mortality data linked to SARS-CoV-2 infection status in UK Biobank is currently unavailable. Future availability of linked hospital outcome and mortality data within UK Biobank will aid in further assessing risk related to SARS-CoV-2 infection.

Despite these limitations, our complementary analytical approaches, including use of the test-negative case-control design, enabled us to triangulate across the different outcomes strengthening the evidence linking a range of exposures to COVID-19 risk.

## Conclusions

Linkage of SARS-CoV-2 test results to UK Biobank enabled us to identify independent associations of demographic, social, health risk, medical and environmental factors with risk of testing positive (confirmed) or negative (suspected) for COVID-19. Of these, male sex, lower educational attainment, non-White ethnicity were also found associated with increased risk of testing positive, within the tested population. Elucidation of the joint and independent effects of these factors represents a high-priority area for further research, which may inform on COVID-19 natural history and suggest possible new avenues to pursue for its prevention.

## Funding

M.C.-H., R.V., M.K.-I. and C.D. acknowledge support from the H2020-EXPANSE project (Horizon 2020 grant No 874627 to RV). M.C.-H, R.V., J.E. and B.B. acknowledge support from Cancer Research UK, Population Research Committee Project grant 'Mechanomics’ (grant No 22184 to MC-H). This work was also supported by EXPOSOME-NL, which is funded through the Gravitation program of the Dutch Ministry of Education, Culture, and Science and the Netherlands Organization for Scientific Research (NWO grant number 024.004.017 to R.V.). B.B. received a PhD studentship from the MRC Centre for Environment and Health. This study was conducted using the UK Biobank resource under application number 19266 granting access to the corresponding UK Biobank biomarkers, and phenotype data. P.E. is Director of the MRC Centre for Environment and Health (MR/L01341X/1, MR/S019669/1). P.E. also acknowledges support from the National Institute for Health Research Imperial Biomedical Research Centre and the NIHR Health Protection Research Units in Environmental Exposures and Health and Chemical and Radiation Threats and Hazards, and the BHF Centre for Research Excellence at Imperial College London (RE/18/4/34215). The funders had no role in the design and conduct of the study; collection, management, analysis, and interpretation of the data; and preparation, review, or approval of this manuscript.

## Supplementary Material

dyaa134_Supplementary_Data
