# Supplementary material for: Risk factors for positive and negative COVID-19 tests: a cautious and in-depth analysis of UK biobank data
Source: Int J Epidemiol. 2020 Aug 20:dyaa134. doi: 10.1093/ije/dyaa134 (PMC7454561; doi:10.1093/ije/dyaa134)
Supplement: dyaa134_Supplementary_Data [file dyaa134_supplementary_data.docx]

Supplementary Information: Risk factors for positive and negative COVID-19 tests: a cautious and in-depth analysis of UK Biobank data

Dr Marc Chadeau-Hyam^a,b, †^, Barbara Bodinier ^a,b,†^, Dr Joshua Elliott^a,b,c,*^, Matthew Whitaker ^a,b,*^, Dr Ioanna Tzoulaki^a,b,d^, Prof Roel Vermeulen^e^, Dr Michelle Kelly-Irving^f,‡^; Dr Cyrille Delpierre ^f,‡^; Prof Paul Elliott^a,b,‡^

^a^ Department of Epidemiology and Biostatistics, School of Public Health, Imperial College London, London, UK

^b^ MRC Centre for Environment and Health, Imperial College, London, UK

^c^ Royal Surrey County Hospital, Guildford, Surrey, GU2 7XX

^d^ Department of Hygiene and Epidemiology, University of Ioannina Medical School, Ioannina, Greece

^e^ Institute for Risk Assessment Sciences (IRAS), Utrecht University, Utrecht, The Netherlands

^f^ UMR LEASP, Université de Toulouse III, UPS, Inserm, Toulouse, France

^†^ Joint first authors

^*^ Equal contribution

^‡^ Joint last authors

# Address for Correspondence:

Dr Marc Chadeau-Hyam and Prof Paul Elliott

Department of Epidemiology and Biostatistics, School of Public Health,

St Mary’s Hospital, Norfolk Place

London, W21PG, UK

E-mail: [m.chadeau@imperial.ac.uk](mailto:m.chadeau@imperial.ac.uk)

E-mail: [p.elliott@imperial.ac.uk](mailto:p.elliott@imperial.ac.uk)

**Supplementary Figure S1.** Comparison of the odds ratio (A) and p-values (B) from the logistic models of the probability of being tested, fitted on the full population (X-axis, up to 4,509 tested *vs.* 483,574 non-tested participants) and on the population excluding healthcare workers (Y-axis, up to 3,823 tested *vs.* 450,244 non-tested participants) for demographics (in grey, N=4), social (brown, N=11), health risk (red, N=7), medical (blue, N=8), and environmental (green, N=4) factors. Effect size estimates (C) and p-values (D) are also compared for the fully adjusted model restricting the population to healthcare workers and comparing the (N=628) tested (X-axis) to the (N=27,789) non tested participants (X-axis), and a random subsample of the same size of non-healthcare workers (Y-axis).

**Supplementary Table S1**. List of UK Biobank Field IDs and their descriptions and corresponding data coding (for categorical variables) that were used to construct the variables in each category (A). Disease codes that were used to define (i) cancer, (ii) cardiovascular disease, (iii) hypertension, (iv) diabetes, (v) respiratory, and (vi) autoimmune disease, based on self-reported information at baseline (UK Biobank fields 20001 and 20002), and linkage to the Hospital Episode Statistics (B).

| Category | Field ID | Description | Data Coding | Variable |
| --- | --- | --- | --- | --- |
| **Demographics** | [34](http://biobank.ndph.ox.ac.uk/showcase/field.cgi?id=34) | [Year of birth](http://biobank.ndph.ox.ac.uk/showcase/field.cgi?id=34) |  | Age (years) |
|  | [52](http://biobank.ndph.ox.ac.uk/showcase/field.cgi?id=52) | [Month of birth](http://biobank.ndph.ox.ac.uk/showcase/field.cgi?id=52) |  |  |
|  | [31](http://biobank.ndph.ox.ac.uk/showcase/field.cgi?id=31) | [Sex](http://biobank.ndph.ox.ac.uk/showcase/field.cgi?id=31) | 9 | Sex |
|  | [21000](http://biobank.ndph.ox.ac.uk/showcase/field.cgi?id=21000) | [Ethnic background](http://biobank.ndph.ox.ac.uk/showcase/field.cgi?id=21000) | 1001 | Ethnicity |
| **Social** | [6138](http://biobank.ndph.ox.ac.uk/showcase/field.cgi?id=6138) | [Qualifications](http://biobank.ndph.ox.ac.uk/showcase/field.cgi?id=6138) | 100305 | Education |
|  | [670](http://biobank.ndph.ox.ac.uk/showcase/field.cgi?id=670) | [Type of accommodation lived in](http://biobank.ndph.ox.ac.uk/showcase/field.cgi?id=670) | 100286 | Type of accommodation |
|  | [680](http://biobank.ndph.ox.ac.uk/showcase/field.cgi?id=680) | [Own or rent accommodation lived in](http://biobank.ndph.ox.ac.uk/showcase/field.cgi?id=680) | 100287 | Own or rent accommodation |
|  | [709](http://biobank.ndph.ox.ac.uk/showcase/field.cgi?id=709) | [Number in household](http://biobank.ndph.ox.ac.uk/showcase/field.cgi?id=709) |  | Number in household |
|  | [738](http://biobank.ndph.ox.ac.uk/showcase/field.cgi?id=738) | [Average total household income before tax](http://biobank.ndph.ox.ac.uk/showcase/field.cgi?id=738) | 100294 | Average household income (GBP) |
|  | [6142](http://biobank.ndph.ox.ac.uk/showcase/field.cgi?id=6142) | [Current employment status](http://biobank.ndph.ox.ac.uk/showcase/field.cgi?id=6142) | 100295 | Occupation |
|  | [132](http://biobank.ndph.ox.ac.uk/showcase/field.cgi?id=132) | [Job code at visit - entered](http://biobank.ndph.ox.ac.uk/showcase/field.cgi?id=132) | 2 |  |
| **Health Risk Factors** | [20116](http://biobank.ndph.ox.ac.uk/showcase/field.cgi?id=20116) | [Smoking status](http://biobank.ndph.ox.ac.uk/showcase/field.cgi?id=20116) | 90 | Smoking status |
|  | [20117](http://biobank.ndph.ox.ac.uk/showcase/field.cgi?id=20117) | [Alcohol drinker status](http://biobank.ndph.ox.ac.uk/showcase/field.cgi?id=20117) | 90 | Alcohol drinker status |
|  | [21001](http://biobank.ndph.ox.ac.uk/showcase/field.cgi?id=21001) | [Body mass index (BMI)](http://biobank.ndph.ox.ac.uk/showcase/field.cgi?id=21001) |  | BMI (kg/m^2^) |
| **Medical** | [20001](http://biobank.ndph.ox.ac.uk/showcase/field.cgi?id=20001) | [Cancer code, self-reported](http://biobank.ndph.ox.ac.uk/showcase/field.cgi?id=20001) | 3 | Cancer |
|  | [20002](http://biobank.ndph.ox.ac.uk/showcase/field.cgi?id=20002) | [Non-cancer illness code, self-reported](http://biobank.ndph.ox.ac.uk/showcase/field.cgi?id=20002) | 6 | Cardiovascular  Hypertension Diabetes  Respiratory  Autoimmune |
|  | [137](http://biobank.ndph.ox.ac.uk/showcase/field.cgi?id=137) | [Number of treatments/medications taken](http://biobank.ndph.ox.ac.uk/showcase/field.cgi?id=137) |  | Number of medications |
| **Environmental** | [24004](http://biobank.ndph.ox.ac.uk/showcase/field.cgi?id=24004) | [Nitrogen oxides air pollution; 2010](http://biobank.ndph.ox.ac.uk/showcase/field.cgi?id=24004) |  | Nitrogen oxides (ug/m^3^) |
|  | [24005](http://biobank.ndph.ox.ac.uk/showcase/field.cgi?id=24005) | [Particulate matter air pollution (pm10); 2010](http://biobank.ndph.ox.ac.uk/showcase/field.cgi?id=24005) |  | PM10 (ug/m^3^) |
|  | [24007](http://biobank.ndph.ox.ac.uk/showcase/field.cgi?id=24007) | [Particulate matter air pollution (pm2.5) absorbance; 2010](http://biobank.ndph.ox.ac.uk/showcase/field.cgi?id=24007) |  | PM2.5 (absorbance/m) |
|  | [24006](http://biobank.ndph.ox.ac.uk/showcase/field.cgi?id=24006) | [Particulate matter air pollution (pm2.5); 2010](http://biobank.ndph.ox.ac.uk/showcase/field.cgi?id=24006) |  | PM2.5 (ug/m^3^) |

A.

| **Cancer** | | | **Cardiovascular** | | | **Hypertension** | | | **Diabetes** | | | **Respiratory** | | | **Autoimmune (1/2)** | | | **Autoimmune (2/2)** | | |
| --- | --- | --- | --- | --- | --- | --- | --- | --- | --- | --- | --- | --- | --- | --- | --- | --- | --- | --- | --- | --- |
| Field 20001 | ICD-10 | ICD-9 | Field 20002 | ICD-10 | ICD-9 | Field 20002 | ICD-10 | ICD-9 | Field 20002 | ICD-10 | ICD-9 | Field 20002 | ICD-10 | ICD-9 | Field 20002 | ICD-10 | ICD-9 | Field 20002 | ICD-10 | ICD-9 |
| Any | C00-C09 | 140-149 | 1066 | I00-I02 | 390-392 | 1065 | I10-I15 | 401-405 | 1220 | E11 | 250.00 | 1111 | J30-J39 | 470-478 | 1222 | D68.3 | 286.5 | 1426 | L14 | 250.93 |
|  | C15-C26 | 150-159 | 1067 | I05-I09 | 393-398 | 1072 |  |  | 1223 | E12 | 250.02 | 1112 | J40-J47 | 480-488 | 1224 | D68.4 | 286.7 | 1435 | L40 | 255.41 |
|  | C30-C39 | 160-165 | 1068 | I20-I25 | 410-414 |  |  |  |  | E13 | 250.10 | 1113 | J60-J70 | 490-496 | 1225 | D68.5 | 289.81 | 1453 | L43 | 340 |
|  | C40-C41 | 170-176 | 1074 | I26-I28 | 415-417 |  |  |  |  | E14 | 250.12 | 1114 | J80-J84 | 500-508 | 1226 | D69.3 | 287.31 | 1461 | L63.9 | 357.81 |
|  | C43-C44 | 179-189 | 1075 | I30-I52 | 420-429 |  |  |  |  |  | 250.20 | 1115 | J85-J86 | 510-519 | 1228 | D86 | 135 | 1462 | L80 | 358 |
|  | C45-C49 | 190-199 | 1076 | I60-I69 | 430-438 |  |  |  |  |  | 250.22 | 1117 | J90-J94 |  | 1234 | D89 | 245.2 | 1463 | L90.0 | 377.3 |
|  | C50 | 200-209 | 1077 | I70-I76 | 440-445 |  |  |  |  |  | 250.30 | 1120 | J95-J99 |  | 1256 | E06.3 | 250.01 | 1464 | M05 | 447.6 |
|  | C51-C58 | 230-234 | 1078 | I77.0-I77.5 | 447.0-447.5 |  |  |  |  |  | 250.32 | 1121 |  |  | 1260 | E10 | 250.03 | 1475 | M06 | 555 |
|  | C60-C63 | 235-238 | 1079 | I77.7-I77.9 | 447.7-447.9 |  |  |  |  |  | 250.40 | 1122 |  |  | 1261 | E27.1 | 250.11 | 1477 | M07 | 556 |
|  | C64-C68 | 239 | 1080 | I78-I79 | 448-449 |  |  |  |  |  | 250.42 | 1123 |  |  | 1313 | E27.2 | 250.13 | 1480 | M08 | 571.6 |
|  | C69-C72 |  | 1081 | I81-I82 | 453 |  |  |  |  |  | 250.50 | 1124 |  |  | 1331 | G35 | 250.21 | 1481 | M30 | 571.42 |
|  | C73-C75 |  | 1082 | I95-I99 |  |  |  |  |  |  | 250.52 | 1125 |  |  | 1345 | G61 | 250.23 | 1506 | M31 | 576.1 |
|  | C76-C80 |  | 1083 |  |  |  |  |  |  |  | 250.60 | 1126 |  |  | 1371 | G70 | 250.31 | 1520 | M32 | 579.0 |
|  | C82-C96 |  | 1086 |  |  |  |  |  |  |  | 250.62 |  |  |  | 1372 | H46 | 250.33 | 1522 | M33 | 694 |
|  | C97 |  | 1087 |  |  |  |  |  |  |  | 250.70 |  |  |  | 1376 | I77.6 | 250.41 | 1549 | M34 | 696 |
|  | D00-D09 |  | 1088 |  |  |  |  |  |  |  | 250.72 |  |  |  | 1377 | K50 | 250.43 | 1550 | M35 | 697.0 |
|  | D37-D48 |  | 1093 |  |  |  |  |  |  |  | 250.80 |  |  |  | 1378 | K51 | 250.51 | 1561 | M45 | 704.01 |
|  |  |  | 1094 |  |  |  |  |  |  |  | 250.82 |  |  |  | 1379 | K74.3 | 250.53 | 1564 | M46.0 | 709.01 |
|  |  |  |  |  |  |  |  |  |  |  | 250.90 |  |  |  | 1380 | K75.4 | 250.61 | 1609 | M46.1 | 701.0 |
|  |  |  |  |  |  |  |  |  |  |  | 250.92 |  |  |  | 1381 | K83.01 | 250.63 | 1661 | M46.2 | 714 |
|  |  |  |  |  |  |  |  |  |  |  |  |  |  |  | 1382 | K90.0 | 250.71 | 1667 | M46.4 | 446 |
|  |  |  |  |  |  |  |  |  |  |  |  |  |  |  | 1383 | L10 | 250.73 |  | M46.8 | 710 |
|  |  |  |  |  |  |  |  |  |  |  |  |  |  |  | 1384 | L11 | 250.81 |  | M46.9 | 720.0 |
|  |  |  |  |  |  |  |  |  |  |  |  |  |  |  | 1397 | L12 | 250.83 |  | N08 | 720.8 |
|  |  |  |  |  |  |  |  |  |  |  |  |  |  |  | 1428 | L13 | 250.91 |  | D51.0 | 281.0 |

B.

**Supplementary Table S2.** Characteristics of the tested population for COVID-19 for participants tested once, twice, and more than twice.

|  |  | **Tested once**  **(N=2,685)** | | **Tested twice**  **(N=1,231)** | | **Tested three times or more**  **(N=593)** | |
| --- | --- | --- | --- | --- | --- | --- | --- |
|  |  | N | Mean (s.d.)  / Proportion | N | Mean (s.d.)  / Proportion | N | Mean (s.d.)  / Proportion |
|  | Test positive | 587 | 21.86% | 448 | 36.39% | 290 | 48.90% |
| **Demographics** | Age (years) | 2,685 | 68.18 (9.01) | 1,231 | 68.97 (8.77) | 593 | 70.01 (8.31) |
|  | Sex |  |  |  |  |  |  |
|  | *Female* | 1,413 | 52.63% | 643 | 52.23% | 252 | 42.50% |
|  | *Male* | 1,272 | 47.37% | 588 | 47.77% | 341 | 57.50% |
|  | Ethnicity |  |  |  |  |  |  |
|  | *White* | 2,454 | 91.88% | 1,091 | 89.13% | 522 | 88.93% |
|  | *Black* | 86 | 3.22% | 58 | 4.74% | 23 | 3.92% |
|  | *Other* | 131 | 4.90% | 75 | 6.13% | 42 | 7.16% |
| **Social** | Education |  |  |  |  |  |  |
|  | *High* | 769 | 29.40% | 329 | 27.49% | 152 | 26.76% |
|  | *Intermediate* | 1,295 | 49.50% | 553 | 46.20% | 260 | 45.77% |
|  | *Low* | 552 | 21.10% | 315 | 26.32% | 156 | 27.46% |
|  | Type of accommodation |  |  |  |  |  |  |
|  | *House* | 2,337 | 88.49% | 1,032 | 85.64% | 457 | 78.93% |
|  | *Flat* | 304 | 11.51% | 173 | 14.36% | 122 | 21.07% |
|  | Own or rent accommodation |  |  |  |  |  |  |
|  | *Own outright* | 1,234 | 47.50% | 574 | 48.40% | 249 | 43.84% |
|  | *Own with a mortgage* | 949 | 36.53% | 406 | 34.23% | 180 | 31.69% |
|  | *Rent* | 415 | 15.97% | 206 | 17.37% | 139 | 24.47% |
|  | Number in household | 2,642 | 2.47 (1.61) | 1,197 | 2.42 (1.50) | 582 | 2.16 (1.09) |
|  | Average household income (GBP) |  |  |  |  |  |  |
|  | *Less than 18,000* | 683 | 30.23% | 327 | 32.70% | 187 | 39.04% |
|  | *18,000 to 30,999* | 548 | 24.26% | 245 | 24.50% | 125 | 26.10% |
|  | *31,000 to 51,999* | 519 | 22.97% | 225 | 22.50% | 90 | 18.79% |
|  | *Greater than 52,000* | 509 | 22.53% | 203 | 20.30% | 77 | 16.08% |
|  | Occupation |  |  |  |  |  |  |
|  | *Unemployed* | 421 | 15.87% | 230 | 18.96% | 133 | 22.70% |
|  | *Employed (Healthcare worker)* | 410 | 15.46% | 165 | 13.60% | 53 | 9.04% |
|  | *Employed (Other)* | 933 | 35.18% | 388 | 31.99% | 176 | 30.03% |
|  | *Retired* | 888 | 33.48% | 430 | 35.45% | 224 | 38.23% |
| **Health Risk Factors** | Smoking status |  |  |  |  |  |  |
|  | *Never* | 1,318 | 49.44% | 599 | 49.10% | 252 | 42.93% |
|  | *Former* | 1,017 | 38.15% | 464 | 38.03% | 237 | 40.37% |
|  | *Current* | 331 | 12.42% | 157 | 12.87% | 98 | 16.70% |
|  | Alcohol drinker status |  |  |  |  |  |  |
|  | *Never* | 156 | 5.83% | 81 | 6.62% | 41 | 6.95% |
|  | *Former* | 140 | 5.24% | 67 | 5.48% | 34 | 5.76% |
|  | *Current* | 2,378 | 88.93% | 1,075 | 87.90% | 515 | 87.29% |
|  | Body Mass Index (kg/m^2^) |  |  |  |  |  |  |
|  | *<25* | 747 | 28.16% | 330 | 27.14% | 144 | 24.66% |
|  | *[25,30[* | 1,115 | 42.03% | 502 | 41.28% | 230 | 39.38% |
|  | *[30,40[* | 711 | 26.80% | 319 | 26.23% | 182 | 31.16% |
|  | *>=40* | 80 | 3.02% | 65 | 5.35% | 28 | 4.79% |
| **Medical** | Cancer |  |  |  |  |  |  |
|  | *No* | 2,172 | 80.89% | 942 | 76.52% | 446 | 75.21% |
|  | *Yes* | 513 | 19.11% | 289 | 23.48% | 147 | 24.79% |
|  | Cardiovascular |  |  |  |  |  |  |
|  | *No* | 1,846 | 68.75% | 787 | 63.93% | 352 | 59.36% |
|  | *Yes* | 839 | 31.25% | 444 | 36.07% | 241 | 40.64% |
|  | Hypertension |  |  |  |  |  |  |
|  | *No* | 1,567 | 58.36% | 644 | 52.32% | 281 | 47.39% |
|  | *Yes* | 1,118 | 41.64% | 587 | 47.68% | 312 | 52.61% |
|  | Diabetes |  |  |  |  |  |  |
|  | *No* | 2,346 | 87.37% | 1,041 | 84.57% | 483 | 81.45% |
|  | *Yes* | 339 | 12.63% | 190 | 15.43% | 110 | 18.55% |
|  | Respiratory |  |  |  |  |  |  |
|  | *No* | 1,931 | 71.92% | 850 | 69.05% | 376 | 63.41% |
|  | *Yes* | 754 | 28.08% | 381 | 30.95% | 217 | 36.59% |
|  | Autoimmune |  |  |  |  |  |  |
|  | *No* | 2,163 | 80.56% | 974 | 79.12% | 470 | 79.26% |
|  | *Yes* | 522 | 19.44% | 257 | 20.88% | 123 | 20.74% |
|  | Number of medications |  |  |  |  |  |  |
|  | *0* | 629 | 23.49% | 238 | 19.37% | 121 | 20.40% |
|  | *1* | 433 | 16.17% | 187 | 15.22% | 70 | 11.80% |
|  | *>1* | 1,616 | 60.34% | 804 | 65.42% | 402 | 67.79% |
| **Environmental** | NO_X_ (ug/m^3^) | 2,645 | 44.99 (15.47) | 1,214 | 47.43 (16.24) | 583 | 48.09 (16.91) |
|  | PM10 (ug/m^3^) | 2,642 | 16.28 (1.82) | 1,213 | 16.53 (1.87) | 583 | 16.43 (1.97) |
|  | PM2.5 (absorbance/m) | 2,642 | 1.20 (0.27) | 1,213 | 1.25 (0.30) | 583 | 1.28 (0.31) |
|  | PM2.5 (ug/m^3^) | 2,642 | 10.07 (1.06) | 1,213 | 10.22 (1.06) | 583 | 10.25 (1.17) |

**Supplementary Table S3**. Results from the univariate logistic models predicting from each covariate separately the risk of (i) being tested for COVID-19 (outcome: tested *vs.* non-tested), (ii) being tested positive for COVID-19 (outcome: tested positive *vs.* non-tested), (iii) being tested negative for COVID-19 (outcome: tested negative *vs.* non-tested), and (iv) being tested positive conditionally on being tested (outcome: tested positive *vs.* tested negative). Effect size estimates are expressed as odds ratios and are represented for demographic covariates, social, health risk, medical and environmental factors.

|  |  | |  | | Tested vs non-tested | | Positive vs. non-tested | | Negative vs. non-tested | | Positive vs. negative | |
| --- | --- | --- | --- | --- | --- | --- | --- | --- | --- | --- | --- | --- |
|  |  | |  | | OR | p-value | OR | p-value | OR | p-value | OR | p-value |
| **Demographics** | Age (years) | |  | | 1.07 [1.04-1.11] | 2.39x10^-6^ | 1.00 [0.95-1.06] | 9.50x10^-1^ | 1.11 [1.07-1.15] | 2.40x10^-8^ | 0.92 [0.87-0.98] | 6.48x10^-3^ |
|  | Sex (ref. Female) | | *Male* | | 1.16 [1.09-1.23] | 6.95x10^-7^ | 1.35 [1.21-1.50] | 6.82x10^-8^ | 1.09 [1.02-1.17] | 1.50x10^-2^ | 1.23 [1.09-1.40] | 1.30x10^-3^ |
|  | Ethnicity (ref. White) | | *Black* | | 2.40 [2.05-2.81] | 4.96x10^-28^ | 3.90 [3.09-4.92] | 2.99x10^-30^ | 1.82 [1.47-2.24] | 2.39x10^-8^ | 2.14 [1.57-2.93] | 1.67x10^-6^ |
|  |  | | *Other* | | 1.51 [1.33-1.72] | 4.30x10^-10^ | 2.13 [1.73-2.62] | 8.76x10^-13^ | 1.27 [1.08-1.49] | 4.78x10^-3^ | 1.68 [1.29-2.18] | 1.20x10^-4^ |
| **Social** | Education (ref. High) | | *Intermediate* | | 1.11 [1.04-1.19] | 3.04x10^-3^ | 1.31 [1.14-1.49] | 9.84x10^-5^ | 1.05 [0.96-1.13] | 2.93x10^-1^ | 1.25 [1.07-1.46] | 5.48x10^-3^ |
|  |  | | *Low* | | 1.60 [1.48-1.74] | 6.79x10^-29^ | 2.03 [1.74-2.37] | 1.44x10^-19^ | 1.46 [1.32-1.61] | 8.57x10^-14^ | 1.40 [1.16-1.68] | 3.34x10^-4^ |
|  | Type of accommodation (ref. House) | | | *Flat* | 1.43 [1.31-1.56] | 5.70x10^-16^ | 1.45 [1.24-1.70] | 3.49x10^-6^ | 1.42 [1.28-1.58] | 2.65x10^-11^ | 1.02 [0.85-1.24] | 8.11x10^-1^ |
|  | Own/Rent (ref. Own outright) | *Own with a mortgage* | | | 1.04 [0.97-1.11] | 2.74x10^-1^ | 1.23 [1.09-1.39] | 1.07x10^-3^ | 0.97 [0.90-1.05] | 4.34x10^-1^ | 1.27 [1.09-1.46] | 1.49x10^-3^ |
|  |  | *Rent* | | | 2.05 [1.89-2.23] | 2.53x10^-63^ | 2.49 [2.14-2.90] | 1.21x10^-32^ | 1.89 [1.71-2.09] | 3.10x10^-35^ | 1.32 [1.10-1.58] | 2.62x10^-3^ |
|  | Number in household | |  | | 0.98 [0.95-1.01] | 1.66x10^-1^ | 1.05 [1.01-1.09] | 2.66x10^-2^ | 0.94 [0.90-0.98] | 2.09x10^-3^ | 1.08 [1.03-1.15] | 4.32x10^-3^ |
|  | Income (GBP) (ref. 18,000 to 30,999) | | *Less than 18,000* | | 1.48 [1.36-1.62] | 3.22x10^-19^ | 1.54 [1.32-1.81] | 9.12x10^-8^ | 1.46 [1.32-1.62] | 4.53x10^-13^ | 1.06 [0.87-1.28] | 5.74x10^-1^ |
|  |  |  | *31,000 to 51,999* | | 0.88 [0.80-0.96] | 5.84x10^-3^ | 0.88 [0.74-1.05] | 1.66x10^-1^ | 0.87 [0.78-0.98] | 1.68x10^-2^ | 1.01 [0.82-1.25] | 9.01x10^-1^ |
|  |  | | *Greater than 52,000* | | 0.84 [0.76-0.92] | 2.21x10^-4^ | 0.76 [0.64-0.91] | 3.50x10^-3^ | 0.86 [0.77-0.97] | 1.10x10^-2^ | 0.88 [0.71-1.09] | 2.49x10^-1^ |
|  | Occupation (ref. Employed (Other)) | | *Healthcare worker* | | 3.62 [3.30-3.98] | 1.58x10^-158^ | 4.15 [3.51-4.89] | 1.03x10^-63^ | 3.41 [3.04-3.82] | 6.26x10^-99^ | 1.22 [1.00-1.49] | 5.35x10^-2^ |
|  |  |  | *Unemployed* | | 1.89 [1.73-2.06] | 5.53x10^-47^ | 1.96 [1.67-2.30] | 9.40x10^-17^ | 1.86 [1.68-2.07] | 3.83x10^-32^ | 1.05 [0.87-1.27] | 6.03x10^-1^ |
|  |  | | *Retired* | | 1.72 [1.60-1.84] | 1.28x10^-49^ | 1.65 [1.45-1.89] | 1.40x10^-13^ | 1.74 [1.60-1.89] | 6.60x10^-38^ | 0.95 [0.81-1.11] | 5.25x10^-1^ |
| **Health Risk Factors** | Smoking status (ref. Never) | | *Former* | | 1.27 [1.20-1.36] | 7.40x10^-14^ | 1.31 [1.17-1.47] | 4.19x10^-6^ | 1.26 [1.17-1.36] | 2.75x10^-9^ | 1.04 [0.91-1.20] | 5.63x10^-1^ |
|  |  | | *Current* | | 1.45 [1.33-1.59] | 1.50x10^-15^ | 1.21 [1.01-1.45] | 3.63x10^-2^ | 1.55 [1.40-1.73] | 4.67x10^-16^ | 0.78 [0.63-0.96] | 2.00x10^-2^ |
|  | Alcohol drinker status (ref. Current) | | *Former* | | 1.60 [1.41-1.83] | 1.65x10^-12^ | 1.80 [1.43-2.27] | 5.29x10^-7^ | 1.52 [1.30-1.78] | 2.05x10^-7^ | 1.18 [0.90-1.56] | 2.38x10^-1^ |
|  |  | | *Never* | | 1.45 [1.28-1.64] | 2.57x10^-9^ | 1.76 [1.43-2.16] | 1.05x10^-7^ | 1.33 [1.14-1.54] | 2.43x10^-4^ | 1.32 [1.03-1.71] | 3.14x10^-2^ |
|  | BMI (kg/m^2^) (ref. <25) | | *[25,30[* | | 1.18 [1.10-1.27] | 6.88x10^-6^ | 1.37 [1.19-1.57] | 9.16x10^-6^ | 1.12 [1.03-1.22] | 1.13x10^-2^ | 1.23 [1.04-1.44] | 1.37x10^-2^ |
|  |  | | *[30,40[* | | 1.48 [1.36-1.60] | 1.40x10^-21^ | 1.88 [1.62-2.18] | 1.06x10^-16^ | 1.34 [1.22-1.47] | 1.92x10^-9^ | 1.40 [1.18-1.67] | 1.63x10^-4^ |
|  |  | | *>=40* | | 2.49 [2.12-2.93] | 7.32x10^-29^ | 2.98 [2.23-3.99] | 2.28x10^-13^ | 2.33 [1.92-2.82] | 5.84x10^-18^ | 1.28 [0.90-1.81] | 1.63x10^-1^ |
| **Medical** | Cancer (ref. No) | | *Yes* | | 1.39 [1.30-1.50] | 1.43x10^-19^ | 1.16 [1.01-1.33] | 4.11x10^-2^ | 1.50 [1.38-1.63] | 3.21x10^-21^ | 0.77 [0.66-0.91] | 1.86x10^-3^ |
|  | Cardiovascular (ref. No) | | *Yes* | | 2.05 [1.93-2.18] | 7.71x10^-114^ | 1.88 [1.67-2.11] | 1.47x10^-26^ | 2.13 [1.98-2.29] | 2.09x10^-90^ | 0.88 [0.77-1.01] | 7.42x10^-2^ |
|  | Hypertension (ref. No) | | *Yes* | | 1.68 [1.58-1.78] | 5.64x10^-66^ | 1.75 [1.57-1.95] | 3.61x10^-24^ | 1.65 [1.53-1.77] | 5.01x10^-44^ | 1.06 [0.94-1.21] | 3.47x10^-1^ |
|  | Diabetes (ref. No) | | *Yes* | | 2.21 [2.03-2.40] | 2.01x10^-75^ | 2.42 [2.08-2.81] | 7.78x10^-31^ | 2.12 [1.92-2.35] | 1.32x10^-47^ | 1.14 [0.95-1.37] | 1.54x10^-1^ |
|  | Respiratory (ref. No) | | *Yes* | | 1.60 [1.50-1.70] | 1.05x10^-46^ | 1.52 [1.35-1.72] | 3.93x10^-12^ | 1.63 [1.51-1.76] | 1.28x10^-36^ | 0.93 [0.81-1.08] | 3.43x10^-1^ |
|  | Autoimmune (ref. No) | | *Yes* | | 1.53 [1.42-1.64] | 1.84x10^-29^ | 1.34 [1.16-1.54] | 5.46x10^-5^ | 1.61 [1.47-1.75] | 2.56x10^-27^ | 0.83 [0.71-0.98] | 2.71x10^-2^ |
|  | Number of medications (ref. 0) | | *1* | | 1.02 [0.92-1.12] | 7.10x10^-1^ | 1.08 [0.91-1.29] | 3.65x10^-1^ | 0.99 [0.88-1.11] | 8.76x10^-1^ | 1.09 [0.89-1.35] | 4.01x10^-1^ |
|  |  | | *>1* | | 1.50 [1.39-1.61] | 8.79x10^-28^ | 1.43 [1.25-1.64] | 1.31x10^-7^ | 1.53 [1.40-1.67] | 7.10x10^-22^ | 0.94 [0.80-1.10] | 4.16x10^-1^ |
| **Environmental** | NO_X_ (ug/m^3^) | |  | | 1.12 [1.09-1.15] | 4.79x10^-18^ | 1.19 [1.15-1.24] | 1.34x10^-17^ | 1.08 [1.05-1.12] | 1.02x10^-6^ | 1.14 [1.07-1.21] | 2.46x10^-5^ |
|  | PM10 (ug/m^3^) | |  | | 1.07 [1.04-1.10] | 1.98x10^-6^ | 1.11 [1.05-1.17] | 1.17x10^-4^ | 1.06 [1.02-1.09] | 1.44x10^-3^ | 1.05 [0.99-1.12] | 1.23x10^-1^ |
|  | PM2.5 (absorbance/m) | |  | | 1.14 [1.11-1.17] | 5.09x10^-22^ | 1.18 [1.13-1.24] | 3.63x10^-12^ | 1.12 [1.09-1.16] | 2.08x10^-12^ | 1.06 [1.00-1.13] | 5.69x10^-2^ |
|  | PM2.5 (ug/m^3^) | |  | | 1.14 [1.11-1.17] | 8.08x10^-21^ | 1.26 [1.20-1.32] | 2.81x10^-20^ | 1.09 [1.06-1.13] | 1.56x10^-7^ | 1.16 [1.09-1.24] | 2.32x10^-6^ |

**Supplementary Table S4.** Characteristics of the tested population for COVID-19 during the first and second part of the UK epidemic. For each variable, differences between participants tested in the first and second part of the UK epidemic are evaluated using a Student’s t-test (for continuous variables) or a chi-squared test (for categorical variables).

|  |  | **Tested between**  **16 March and 9 April 2020**  **(N=1,347)** | | **Tested between**  **10 April and 18 May 2020**  **(N=3,162)** | | p-value  (first part *vs.* second part) |
| --- | --- | --- | --- | --- | --- | --- |
|  |  | N | Mean (s.d.)  / Proportion | N | Mean (s.d.)  / Proportion |  |
| **Demographics** | Age (years) | 1,347 | 69.28 (8.66) | 3,162 | 68.36 (8.95) | 1.23x10^-3^ |
|  | Sex |  |  |  |  | 5.47x10^-5^ |
|  | *Female* | 627 | 46.55% | 1,681 | 53.16% |  |
|  | *Male* | 720 | 53.45% | 1,481 | 46.84% |  |
|  | Ethnicity |  |  |  |  | 2.51x10^-3^ |
|  | *White* | 1,188 | 88.86% | 2,879 | 91.54% |  |
|  | *Black* | 69 | 5.16% | 98 | 3.12% |  |
|  | *Other* | 80 | 5.98% | 168 | 5.34% |  |
| **Social** | Education |  |  |  |  | 7.49x10^-4^ |
|  | *High* | 370 | 28.42% | 880 | 28.58% |  |
|  | *Intermediate* | 582 | 44.70% | 1,526 | 49.56% |  |
|  | *Low* | 350 | 26.88% | 673 | 21.86% |  |
|  | Type of accommodation |  |  |  |  | 3.27x10^-2^ |
|  | *House* | 1,116 | 84.74% | 2,710 | 87.19% |  |
|  | *Flat* | 201 | 15.26% | 398 | 12.81% |  |
|  | Own or rent accommodation |  |  |  |  | 3.77x10^-1^ |
|  | *Own outright* | 604 | 46.46% | 1,453 | 47.61% |  |
|  | *Own with a mortgage* | 453 | 34.85% | 1,082 | 35.45% |  |
|  | *Rent* | 243 | 18.69% | 517 | 16.94% |  |
|  | Number in household | 1,315 | 2.38 (1.26) | 3,106 | 2.43 (1.62) | 2.84x10^-1^ |
|  | Average household income (GBP) |  |  |  |  | 5.57x10^-2^ |
|  | *Less than 18,000* | 389 | 35.17% | 808 | 30.70% |  |
|  | *18,000 to 30,999* | 258 | 23.33% | 660 | 25.08% |  |
|  | *31,000 to 51,999* | 230 | 20.80% | 604 | 22.95% |  |
|  | *Greater than 52,000* | 229 | 20.71% | 560 | 21.28% |  |
|  | Occupation |  |  |  |  | 3.40x10^-2^ |
|  | *Unemployed* | 258 | 19.44% | 526 | 16.84% |  |
|  | *Employed (Healthcare worker)* | 168 | 12.66% | 460 | 14.72% |  |
|  | *Employed (Other)* | 425 | 32.03% | 1,072 | 34.31% |  |
|  | *Retired* | 476 | 35.87% | 1,066 | 34.12% |  |
| **Health Risk Factors** | Smoking status |  |  |  |  | 6.70x10^-3^ |
|  | *Never* | 599 | 44.87% | 1,570 | 50.03% |  |
|  | *Former* | 550 | 41.20% | 1,168 | 37.22% |  |
|  | *Current* | 186 | 13.93% | 400 | 12.75% |  |
|  | Alcohol drinker status |  |  |  |  | 7.32x10^-1^ |
|  | *Never* | 78 | 5.82% | 200 | 6.36% |  |
|  | *Former* | 75 | 5.59% | 166 | 5.28% |  |
|  | *Current* | 1,188 | 88.59% | 2,780 | 88.37% |  |
|  | Body Mass Index (kg/m^2^) |  |  |  |  | 1.36x10^-1^ |
|  | *<25* | 336 | 25.42% | 885 | 28.27% |  |
|  | *[25,30[* | 550 | 41.60% | 1,297 | 41.42% |  |
|  | *[30,40[* | 377 | 28.52% | 835 | 26.67% |  |
|  | *>=40* | 59 | 4.46% | 114 | 3.64% |  |
| **Medical** | Cancer |  |  |  |  | 2.64x10^-1^ |
|  | *No* | 1,049 | 77.88% | 2,511 | 79.41% |  |
|  | *Yes* | 298 | 22.12% | 651 | 20.59% |  |
|  | Cardiovascular |  |  |  |  | 2.66x10^-2^ |
|  | *No* | 859 | 63.77% | 2,126 | 67.24% |  |
|  | *Yes* | 488 | 36.23% | 1,036 | 32.76% |  |
|  | Hypertension |  |  |  |  | 1.30x10^-2^ |
|  | *No* | 706 | 52.41% | 1,786 | 56.48% |  |
|  | *Yes* | 641 | 47.59% | 1,376 | 43.52% |  |
|  | Diabetes |  |  |  |  | 4.78x10^-1^ |
|  | *No* | 1,148 | 85.23% | 2,722 | 86.08% |  |
|  | *Yes* | 199 | 14.77% | 440 | 13.92% |  |
|  | Respiratory |  |  |  |  | 7.23x10^-4^ |
|  | *No* | 895 | 66.44% | 2,262 | 71.54% |  |
|  | *Yes* | 452 | 33.56% | 900 | 28.46% |  |
|  | Autoimmune |  |  |  |  | 6.23x10^-1^ |
|  | *No* | 1,071 | 79.51% | 2,536 | 80.20% |  |
|  | *Yes* | 276 | 20.49% | 626 | 19.80% |  |
|  | Number of medications |  |  |  |  | 2.05x10^-3^ |
|  | *0* | 266 | 19.79% | 722 | 22.88% |  |
|  | *1* | 183 | 13.62% | 507 | 16.06% |  |
|  | *>1* | 895 | 66.59% | 1,927 | 61.06% |  |
| **Environmental** | NO_X_ (ug/m^3^) | 1,327 | 47.28 (16.21) | 3,115 | 45.55 (15.78) | 1.04x10^-3^ |
|  | PM10 (ug/m^3^) | 1,327 | 16.43 (1.85) | 3,111 | 16.34 (1.86) | 1.46x10^-1^ |
|  | PM2.5 (absorbance/m) | 1,327 | 1.24 (0.29) | 3,111 | 1.22 (0.29) | 1.76x10^-2^ |
|  | PM2.5 (ug/m^3^) | 1,327 | 10.22 (1.09) | 3,111 | 10.10 (1.07) | 1.61x10^-3^ |

**Supplementary Table S5.** Characteristics of the tested population for COVID-19 who were tested as in- or outpatients. For each variable, the differences between participants tested as inpatients or outpatients are evaluated using a Student’s t-test (for continuous variables) or a chi-squared test (for categorical variables).

|  |  | **Outpatients**  **(N=1,323)** | | **Inpatients**  **(N=3,186)** | | p-value (Tested *vs.* non-tested) |
| --- | --- | --- | --- | --- | --- | --- |
|  |  | N | Mean (s.d.)  / Proportion | N | Mean (s.d.)  / Proportion |  |
| **Demographics** | Age (years) | 1,323 | 66.27 (9.42) | 3,186 | 69.62 (8.45) | 2.37x10^-28^ |
|  | Sex |  |  |  |  | 1.55x10^-9^ |
|  | *Female* | 770 | 58.20% | 1,538 | 48.27% |  |
|  | *Male* | 553 | 41.80% | 1,648 | 51.73% |  |
|  | Ethnicity |  |  |  |  | 4.30x10^-9^ |
|  | *White* | 1,139 | 86.62% | 2,928 | 92.45% |  |
|  | *Black* | 75 | 5.70% | 92 | 2.90% |  |
|  | *Other* | 101 | 7.68% | 147 | 4.64% |  |
| **Social** | Education |  |  |  |  | 1.26x10^-10^ |
|  | *High* | 417 | 32.30% | 833 | 26.96% |  |
|  | *Intermediate* | 657 | 50.89% | 1,451 | 46.96% |  |
|  | *Low* | 217 | 16.81% | 806 | 26.08% |  |
|  | Type of accommodation |  |  |  |  | 1.73x10^-2^ |
|  | *House* | 1,098 | 84.53% | 2,728 | 87.27% |  |
|  | *Flat* | 201 | 15.47% | 398 | 12.73% |  |
|  | Own or rent accommodation |  |  |  |  | 1.12x10^-5^ |
|  | *Own outright* | 533 | 41.90% | 1,524 | 49.48% |  |
|  | *Own with a mortgage* | 509 | 40.02% | 1,026 | 33.31% |  |
|  | *Rent* | 230 | 18.08% | 530 | 17.21% |  |
|  | Number in household | 1,295 | 2.58 (1.60) | 3,126 | 2.35 (1.48) | 8.32x10^-6^ |
|  | Average household income (GBP) |  |  |  |  | 4.65x10^-6^ |
|  | *Less than 18,000* | 303 | 27.42% | 894 | 33.95% |  |
|  | *18,000 to 30,999* | 260 | 23.53% | 658 | 24.99% |  |
|  | *31,000 to 51,999* | 258 | 23.35% | 576 | 21.88% |  |
|  | *Greater than 52,000* | 284 | 25.70% | 505 | 19.18% |  |
|  | Occupation |  |  |  |  | 2.97x10^-21^ |
|  | *Unemployed* | 222 | 17.04% | 562 | 17.85% |  |
|  | *Employed (Healthcare worker)* | 279 | 21.41% | 349 | 11.09% |  |
|  | *Employed (Other)* | 446 | 34.23% | 1,051 | 33.39% |  |
|  | *Retired* | 356 | 27.32% | 1,186 | 37.67% |  |
| **Health Risk Factors** | Smoking status |  |  |  |  | 2.23x10^-6^ |
|  | *Never* | 715 | 54.37% | 1,454 | 46.04% |  |
|  | *Former* | 452 | 34.37% | 1,266 | 40.09% |  |
|  | *Current* | 148 | 11.25% | 438 | 13.87% |  |
|  | Alcohol drinker status |  |  |  |  | 5.58x10^-1^ |
|  | *Never* | 87 | 6.61% | 191 | 6.03% |  |
|  | *Former* | 65 | 4.94% | 176 | 5.55% |  |
|  | *Current* | 1,165 | 88.46% | 2,803 | 88.42% |  |
|  | Body Mass Index (kg/m^2^) |  |  |  |  | 3.01x10^-3^ |
|  | *<25* | 409 | 31.20% | 812 | 25.84% |  |
|  | *[25,30[* | 524 | 39.97% | 1,323 | 42.11% |  |
|  | *[30,40[* | 333 | 25.40% | 879 | 27.98% |  |
|  | *>=40* | 45 | 3.43% | 128 | 4.07% |  |
| **Medical** | Cancer |  |  |  |  | 4.22x10^-4^ |
|  | *No* | 1,089 | 82.31% | 2,471 | 77.56% |  |
|  | *Yes* | 234 | 17.69% | 715 | 22.44% |  |
|  | Cardiovascular |  |  |  |  | 7.88x10^-8^ |
|  | *No* | 954 | 72.11% | 2,031 | 63.75% |  |
|  | *Yes* | 369 | 27.89% | 1,155 | 36.25% |  |
|  | Hypertension |  |  |  |  | 8.35x10^-10^ |
|  | *No* | 825 | 62.36% | 1,667 | 52.32% |  |
|  | *Yes* | 498 | 37.64% | 1,519 | 47.68% |  |
|  | Diabetes |  |  |  |  | 6.78x10^-6^ |
|  | *No* | 1,184 | 89.49% | 2,686 | 84.31% |  |
|  | *Yes* | 139 | 10.51% | 500 | 15.69% |  |
|  | Respiratory |  |  |  |  | 1.25x10^-3^ |
|  | *No* | 972 | 73.47% | 2,185 | 68.58% |  |
|  | *Yes* | 351 | 26.53% | 1,001 | 31.42% |  |
|  | Autoimmune |  |  |  |  | 2.38x10^-3^ |
|  | *No* | 1,096 | 82.84% | 2,511 | 78.81% |  |
|  | *Yes* | 227 | 17.16% | 675 | 21.19% |  |
|  | Number of medications |  |  |  |  | 2.35x10^-8^ |
|  | *0* | 358 | 27.10% | 630 | 19.82% |  |
|  | *1* | 216 | 16.35% | 474 | 14.91% |  |
|  | *>1* | 747 | 56.55% | 2,075 | 65.27% |  |
| **Environmental** | NO_X_ (ug/m^3^) | 1,294 | 46.19 (16.41) | 3,148 | 46.01 (15.73) | 7.47x10^-1^ |
|  | PM10 (ug/m^3^) | 1,294 | 16.39 (1.72) | 3,144 | 16.36 (1.91) | 6.18x10^-1^ |
|  | PM2.5 (absorbance/m) | 1,294 | 1.23 (0.28) | 3,144 | 1.22 (0.29) | 2.12x10^-1^ |
|  | PM2.5 (ug/m^3^) | 1,294 | 10.11 (1.08) | 3,144 | 10.15 (1.08) | 3.59x10^-1^ |

**Supplementary Table S6.** Characteristics of the population tested for COVID-19, comparing healthcare workers with the rest of the participants (including unemployed, retired, or employed with another job). For each variable, the difference between the tested health workers and the rest of the tested participants is evaluated using a Student’s t-test (for continuous variables) or a chi-squared test (for categorical variables).

|  |  | **Non-healthcare-worker**  **(N=3,823)** | | **Healthcare worker**  **(N=628)** | | p-value (Tested *vs.* non-tested) |
| --- | --- | --- | --- | --- | --- | --- |
|  |  | N | Mean (s.d.)  / Proportion | N | Mean (s.d.)  / Proportion |  |
| **Demographics** | Age (years) | 3,823 | 70.06 (8.42) | 628 | 60.09 (6.41) | 1.44x10^-172^ |
|  | Sex |  |  |  |  | 2.03x10^-43^ |
|  | *Female* | 1,800 | 47.08% | 483 | 76.91% |  |
|  | *Male* | 2,023 | 52.92% | 145 | 23.09% |  |
|  | Ethnicity |  |  |  |  | 1.26x10^-10^ |
|  | *White* | 3,506 | 92.07% | 526 | 83.76% |  |
|  | *Black* | 126 | 3.31% | 39 | 6.21% |  |
|  | *Other* | 176 | 4.62% | 63 | 10.03% |  |
| **Social** | Education |  |  |  |  | 3.45x10^-47^ |
|  | *High* | 949 | 25.51% | 290 | 46.85% |  |
|  | *Intermediate* | 1,779 | 47.82% | 311 | 50.24% |  |
|  | *Low* | 992 | 26.67% | 18 | 2.91% |  |
|  | Type of accommodation |  |  |  |  | 2.35x10^-1^ |
|  | *House* | 3,243 | 86.32% | 551 | 88.16% |  |
|  | *Flat* | 514 | 13.68% | 74 | 11.84% |  |
|  | Own or rent accommodation |  |  |  |  | 1.89x10^-64^ |
|  | *Own outright* | 1,919 | 51.88% | 124 | 20.16% |  |
|  | *Own with a mortgage* | 1,125 | 30.41% | 400 | 65.04% |  |
|  | *Rent* | 655 | 17.71% | 91 | 14.80% |  |
|  | Number in household | 3,753 | 2.31 (1.43) | 626 | 3.07 (1.87) | 4.62x10^-21^ |
|  | Average household income (GBP) |  |  |  |  | 6.92x10^-48^ |
|  | *Less than 18,000* | 1,124 | 35.81% | 59 | 10.30% |  |
|  | *18,000 to 30,999* | 790 | 25.17% | 122 | 21.29% |  |
|  | *31,000 to 51,999* | 666 | 21.22% | 166 | 28.97% |  |
|  | *Greater than 52,000* | 559 | 17.81% | 226 | 39.44% |  |
| **Health Risk Factors** | Smoking status |  |  |  |  | 1.91x10^-6^ |
|  | *Never* | 1,779 | 46.78% | 360 | 57.78% |  |
|  | *Former* | 1,516 | 39.86% | 192 | 30.82% |  |
|  | *Current* | 508 | 13.36% | 71 | 11.40% |  |
|  | Alcohol drinker status |  |  |  |  | 1.21x10^-3^ |
|  | *Never* | 214 | 5.61% | 53 | 8.45% |  |
|  | *Former* | 216 | 5.66% | 20 | 3.19% |  |
|  | *Current* | 3,383 | 88.72% | 554 | 88.36% |  |
|  | Body Mass Index (kg/m^2^) |  |  |  |  | 8.60x10^-6^ |
|  | *<25* | 987 | 26.17% | 223 | 35.62% |  |
|  | *[25,30[* | 1,584 | 41.99% | 242 | 38.66% |  |
|  | *[30,40[* | 1,049 | 27.81% | 145 | 23.16% |  |
|  | *>=40* | 152 | 4.03% | 16 | 2.56% |  |
| **Medical** | Cancer |  |  |  |  | 1.75x10^-9^ |
|  | *No* | 2,966 | 77.58% | 554 | 88.22% |  |
|  | *Yes* | 857 | 22.42% | 74 | 11.78% |  |
|  | Cardiovascular |  |  |  |  | 8.89x10^-32^ |
|  | *No* | 2,408 | 62.99% | 546 | 86.94% |  |
|  | *Yes* | 1,415 | 37.01% | 82 | 13.06% |  |
|  | Hypertension |  |  |  |  | 1.52x10^-37^ |
|  | *No* | 1,968 | 51.48% | 496 | 78.98% |  |
|  | *Yes* | 1,855 | 48.52% | 132 | 21.02% |  |
|  | Diabetes |  |  |  |  | 9.37x10^-12^ |
|  | *No* | 3,228 | 84.44% | 595 | 94.75% |  |
|  | *Yes* | 595 | 15.56% | 33 | 5.25% |  |
|  | Respiratory |  |  |  |  | 5.64x10^-10^ |
|  | *No* | 2,609 | 68.24% | 506 | 80.57% |  |
|  | *Yes* | 1,214 | 31.76% | 122 | 19.43% |  |
|  | Autoimmune |  |  |  |  | 2.59x10^-8^ |
|  | *No* | 3,009 | 78.71% | 555 | 88.38% |  |
|  | *Yes* | 814 | 21.29% | 73 | 11.62% |  |
|  | Number of medications |  |  |  |  | 4.33x10^-24^ |
|  | *0* | 760 | 19.92% | 217 | 34.55% |  |
|  | *1* | 552 | 14.47% | 133 | 21.18% |  |
|  | *>1* | 2,504 | 65.62% | 278 | 44.27% |  |
| **Environmental** | NO_X_ (ug/m^3^) | 3,767 | 45.79 (15.40) | 618 | 47.65 (18.57) | 1.81x10^-2^ |
|  | PM10 (ug/m^3^) | 3,764 | 16.36 (1.85) | 617 | 16.40 (1.87) | 6.13x10^-1^ |
|  | PM2.5 (absorbance/m) | 3,764 | 1.22 (0.28) | 617 | 1.25 (0.30) | 3.86x10^-2^ |
|  | PM2.5 (ug/m^3^) | 3,764 | 10.12 (1.07) | 617 | 10.20 (1.15) | 1.08x10^-1^ |

**Supplementary Table S7.** Odds ratio [95% confidence intervals], and p-values from the logistic models for the probability of being tested for COVID-19 (A, outcome: tested *vs.* non-tested), and of testing positive conditionally on being tested (B, outcome: positive vs. negative). Results are represented for sequentially adjusted models, where benchmark predictors are defined as demographic descriptors (D, Model 1), and models are additionally adjusted for social (S, Model 2), health risk (HRF, Model 3), medical (M, Model 4), and environmental factors (E, Model 5).

A.

|  |  | | | | | |  | | Model 1 | | Model 2 | | Model 3 | | Model 4 | | Model 5 | |
| --- | --- | --- | --- | --- | --- | --- | --- | --- | --- | --- | --- | --- | --- | --- | --- | --- | --- | --- |
|  |  | | | | | |  | | OR | p-value | OR | p-value | OR | p-value | OR | p-value | OR | p-value |
| D | Age (years) | | | | | |  | | 1.08 [1.04-1.12] | 1.48x10^-5^ | 1.04 [0.99-1.09] | 1.47x10^-1^ | 1.02 [0.98-1.07] | 3.53x10^-1^ | 0.93 [0.88-0.97] | 2.17x10^-3^ | 0.93 [0.88-0.97] | 2.81x10^-3^ |
|  | Sex (ref. Female) | | | | | | *Male* | | 1.05 [1.01-1.08] | 4.72x10^-3^ | 1.14 [1.10-1.18] | 3.07x10^-13^ | 1.13 [1.09-1.17] | 5.29x10^-11^ | 1.10 [1.06-1.14] | 4.10x10^-7^ | 1.10 [1.06-1.14] | 3.43x10^-7^ |
|  | Ethnicity (ref. White) | | | | | | *Black* | | 1.11 [1.09-1.14] | 2.10x10^-20^ | 1.06 [1.04-1.09] | 1.32x10^-6^ | 1.06 [1.03-1.08] | 8.77x10^-6^ | 1.06 [1.03-1.08] | 4.20x10^-6^ | 1.05 [1.02-1.08] | 7.55x10^-5^ |
|  |  | | | | | | *Other* | | 1.07 [1.04-1.10] | 3.47x10^-6^ | 1.03 [1.00-1.07] | 2.27x10^-2^ | 1.03 [1.00-1.06] | 5.74x10^-2^ | 1.02 [0.99-1.06] | 1.21x10^-1^ | 1.02 [0.99-1.05] | 2.94x10^-1^ |
| S | Education (ref. High) | | | | | *Intermediate* | | |  |  | 1.04 [1.00-1.09] | 3.46x10^-2^ | 1.03 [0.99-1.07] | 1.73x10^-1^ | 1.02 [0.98-1.06] | 3.28x10^-1^ | 1.03 [0.98-1.07] | 2.18x10^-1^ |
|  |  | | | | | | *Low* | |  |  | 1.13 [1.08-1.17] | 1.52x10^-9^ | 1.10 [1.06-1.15] | 5.91x10^-7^ | 1.08 [1.04-1.13] | 3.71x10^-5^ | 1.09 [1.05-1.13] | 2.84x10^-5^ |
|  | Type of accommodation (ref. House) | | | | | | | *Flat* |  |  | 1.05 [1.02-1.09] | 2.55x10^-3^ | 1.05 [1.02-1.09] | 2.49x10^-3^ | 1.05 [1.02-1.09] | 1.79x10^-3^ | 1.03 [1.00-1.07] | 6.06x10^-2^ |
|  | Own/Rent (ref. Own outright) | *Own with a mortgage* | | | | | | |  |  | 1.14 [1.10-1.19] | 3.65x10^-10^ | 1.13 [1.08-1.18] | 1.96x10^-8^ | 1.11 [1.07-1.16] | 9.24x10^-7^ | 1.11 [1.07-1.16] | 9.60x10^-7^ |
|  |  | | | | | | *Rent* | |  |  | 1.15 [1.11-1.19] | 4.02x10^-15^ | 1.12 [1.08-1.16] | 1.77x10^-10^ | 1.10 [1.06-1.13] | 3.39x10^-7^ | 1.09 [1.06-1.13] | 6.30x10^-7^ |
|  | Number in household | | | | | |  | |  |  | 1.05 [1.02-1.08] | 1.10x10^-3^ | 1.05 [1.02-1.08] | 8.09x10^-4^ | 1.05 [1.02-1.08] | 5.64x10^-4^ | 1.05 [1.02-1.08] | 6.17x10^-4^ |
|  | Income (GBP) (ref. 18,000 to 30,999) | | *Less than 18,000* | | | | | |  |  | 1.08 [1.04-1.13] | 1.17x10^-4^ | 1.07 [1.03-1.12] | 5.36x10^-4^ | 1.06 [1.02-1.10] | 6.13x10^-3^ | 1.06 [1.01-1.10] | 8.16x10^-3^ |
|  |  | *31,000 to 51,999* | | | | | | |  |  | 0.97 [0.93-1.02] | 2.02x10^-1^ | 0.98 [0.93-1.02] | 2.94x10^-1^ | 0.98 [0.94-1.03] | 4.15x10^-1^ | 0.98 [0.94-1.03] | 4.56x10^-1^ |
|  |  | *Greater than 52,000* | | | | | | |  |  | 0.96 [0.91-1.01] | 7.95x10^-2^ | 0.97 [0.93-1.02] | 2.26x10^-1^ | 0.98 [0.94-1.03] | 4.33x10^-1^ | 0.98 [0.94-1.03] | 4.67x10^-1^ |
|  | Occupation (ref. Employed (Other)) | | | *Healthcare worker* | | | | |  |  | 1.40 [1.37-1.44] | 3.99x10^-150^ | 1.40 [1.36-1.44] | 1.45x10^-148^ | 1.39 [1.35-1.42] | 4.21x10^-142^ | 1.39 [1.35-1.43] | 1.84x10^-142^ |
|  |  | | | *Unemployed* | | | | |  |  | 1.17 [1.13-1.21] | 3.11x10^-17^ | 1.16 [1.12-1.20] | 2.66x10^-15^ | 1.12 [1.08-1.16] | 9.60x10^-10^ | 1.12 [1.08-1.16] | 7.71x10^-10^ |
|  |  | | | *Retired* | | | | |  |  | 1.25 [1.20-1.32] | 1.45x10^-20^ | 1.26 [1.20-1.32] | 8.28x10^-21^ | 1.23 [1.17-1.29] | 1.68x10^-17^ | 1.24 [1.18-1.30] | 3.57x10^-18^ |
| HRF | Smoking status (ref. Never) | | | | | | *Former* | |  |  |  |  | 1.10 [1.06-1.14] | 2.49x10^-7^ | 1.08 [1.04-1.11] | 5.56x10^-5^ | 1.07 [1.04-1.11] | 1.09x10^-4^ |
|  |  | | | | | | *Current* | |  |  |  |  | 1.07 [1.04-1.11] | 3.39x10^-5^ | 1.05 [1.02-1.09] | 1.85x10^-3^ | 1.05 [1.02-1.09] | 3.57x10^-3^ |
|  | Alcohol drinker status (ref. Current) | | | | | | *Former* | |  |  |  |  | 1.05 [1.02-1.08] | 7.96x10^-4^ | 1.03 [1.00-1.06] | 2.56x10^-2^ | 1.03 [1.00-1.06] | 2.81x10^-2^ |
|  |  | | | | | | *Never* | |  |  |  |  | 1.05 [1.02-1.08] | 6.09x10^-4^ | 1.04 [1.01-1.07] | 4.55x10^-3^ | 1.04 [1.01-1.07] | 5.67x10^-3^ |
|  | BMI (kg/m^2^) (ref. <25) | | | | | | *[25.30[* | |  |  |  |  | 1.05 [1.01-1.09] | 2.31x10^-2^ | 1.03 [0.98-1.07] | 2.21x10^-1^ | 1.03 [0.99-1.07] | 2.05x10^-1^ |
|  |  | | | | | | *[30,40[* | |  |  |  |  | 1.10 [1.06-1.15] | 6.35x10^-7^ | 1.03 [0.99-1.08] | 1.01x10^-1^ | 1.03 [0.99-1.08] | 9.43x10^-2^ |
|  |  | | | | | | *>=40* | |  |  |  |  | 1.09 [1.07-1.12] | 6.08x10^-12^ | 1.05 [1.02-1.08] | 5.61x10^-4^ | 1.05 [1.02-1.08] | 5.72x10^-4^ |
| M | Cancer (ref. No) | | | | | | *Yes* | |  |  |  |  |  |  | 1.09 [1.05-1.12] | 2.31x10^-7^ | 1.09 [1.05-1.12] | 2.24x10^-7^ |
|  | Cardiovascular (ref. No) | | | | | | *Yes* | |  |  |  |  |  |  | 1.19 [1.15-1.22] | 4.10x10^-26^ | 1.19 [1.15-1.22] | 4.04x10^-26^ |
|  | Hypertension (ref. No) | | | | | | *Yes* | |  |  |  |  |  |  | 1.07 [1.03-1.11] | 2.38x10^-4^ | 1.07 [1.03-1.11] | 2.98x10^-4^ |
|  | Diabetes (ref. No) | | | | | | *Yes* | |  |  |  |  |  |  | 1.08 [1.05-1.11] | 6.89x10^-9^ | 1.08 [1.05-1.11] | 9.52x10^-9^ |
|  | Respiratory (ref. No) | | | | *Yes* | | | |  |  |  |  |  |  | 1.10 [1.07-1.13] | 2.37x10^-9^ | 1.10 [1.06-1.13] | 3.06x10^-9^ |
|  | Autoimmune (ref. No) | | | | *Yes* | | | |  |  |  |  |  |  | 1.08 [1.04-1.11] | 1.87x10^-6^ | 1.08 [1.04-1.11] | 1.98x10^-6^ |
|  | Number of medications (ref. 0) | | | | *1* | | | |  |  |  |  |  |  | 0.99 [0.95-1.03] | 6.44x10^-1^ | 0.99 [0.95-1.03] | 6.65x10^-1^ |
|  |  | | | | | | | *>1* |  |  |  |  |  |  | 1.02 [0.97-1.06] | 5.02x10^-1^ | 1.02 [0.97-1.07] | 4.61x10^-1^ |
| E | NO_X_ (ug/m^3^) | | | | | |  | |  |  |  |  |  |  |  |  | 1.02 [0.96-1.08] | 6.08x10^-1^ |
|  | PM10 (ug/m^3^) | | | | | |  | |  |  |  |  |  |  |  |  | 0.97 [0.93-1.01] | 1.89x10^-1^ |
|  | PM2.5 (absorbance/m) | | | | | |  | |  |  |  |  |  |  |  |  | 1.08 [1.03-1.13] | 7.99x10^-4^ |
|  | PM2.5 (ug/m^3^) | | | | | |  | |  |  |  |  |  |  |  |  | 1.02 [0.96-1.08] | 6.24x10^-1^ |

B.

|  |  | | | | | |  | | Model 1 | | Model 2 | | Model 3 | | Model 4 | | Model 5 | |
| --- | --- | --- | --- | --- | --- | --- | --- | --- | --- | --- | --- | --- | --- | --- | --- | --- | --- | --- |
|  |  | | | | | |  | | OR | p-value | OR | p-value | OR | p-value | OR | p-value | OR | p-value |
| D | Age (years) | | | | | |  | | 0.88 [0.82-0.95] | 1.14x10^-3^ | 0.88 [0.79-0.99] | 2.95x10^-2^ | 0.87 [0.78-0.98] | 1.80x10^-2^ | 0.90 [0.79-1.01] | 7.02x10^-2^ | 0.90 [0.80-1.02] | 8.87x10^-2^ |
|  | Sex (ref. Female) | | | | | | *Male* | | 1.13 [1.05-1.22] | 1.30x10^-3^ | 1.15 [1.07-1.25] | 3.33x10^-4^ | 1.14 [1.06-1.24] | 9.46x10^-4^ | 1.13 [1.04-1.23] | 2.85x10^-3^ | 1.13 [1.04-1.23] | 3.20x10^-3^ |
|  | Ethnicity (ref. White) | | | | | | *Black* | | 1.12 [1.05-1.20] | 7.87x10^-4^ | 1.12 [1.05-1.20] | 1.10x10^-3^ | 1.12 [1.04-1.20] | 1.74x10^-3^ | 1.11 [1.04-1.19] | 3.61x10^-3^ | 1.11 [1.03-1.19] | 5.15x10^-3^ |
|  |  | | | | | | *Other* | | 1.15 [1.07-1.23] | 6.06x10^-5^ | 1.15 [1.07-1.23] | 9.36x10^-5^ | 1.16 [1.07-1.25] | 1.29x10^-4^ | 1.15 [1.06-1.24] | 3.36x10^-4^ | 1.14 [1.06-1.23] | 6.42x10^-4^ |
| S | Education (ref. High) | | | | | *Intermediate* | | |  |  | 1.15 [1.05-1.26] | 3.09x10^-3^ | 1.14 [1.04-1.26] | 4.54x10^-3^ | 1.15 [1.05-1.26] | 3.44x10^-3^ | 1.15 [1.05-1.27] | 2.86x10^-3^ |
|  |  | | | | | | *Low* | |  |  | 1.24 [1.12-1.37] | 1.79x10^-5^ | 1.24 [1.12-1.37] | 2.82x10^-5^ | 1.24 [1.12-1.37] | 3.06x10^-5^ | 1.23 [1.11-1.36] | 4.69x10^-5^ |
|  | Type of accommodation (ref. House) | | | | | | | *Flat* |  |  | 0.98 [0.90-1.06] | 5.81x10^-1^ | 0.98 [0.90-1.07] | 7.13x10^-1^ | 0.98 [0.90-1.07] | 7.28x10^-1^ | 0.97 [0.88-1.06] | 4.66x10^-1^ |
|  | Own/Rent (ref. Own outright) | *Own with a mortgage* | | | | | | |  |  | 1.10 [1.00-1.22] | 4.22x10^-2^ | 1.10 [1.00-1.21] | 5.14x10^-2^ | 1.10 [0.99-1.21] | 6.47x10^-2^ | 1.09 [0.99-1.20] | 9.36x10^-2^ |
|  |  | | | | | | *Rent* | |  |  | 1.01 [0.92-1.10] | 9.14x10^-1^ | 1.00 [0.91-1.10] | 9.95x10^-1^ | 1.00 [0.91-1.10] | 9.95x10^-1^ | 0.99 [0.89-1.08] | 7.62x10^-1^ |
|  | Number in household | | | | | |  | |  |  | 1.04 [0.96-1.13] | 3.14x10^-1^ | 1.04 [0.96-1.13] | 3.29x10^-1^ | 1.04 [0.96-1.12] | 3.87x10^-1^ | 1.04 [0.96-1.13] | 2.96x10^-1^ |
|  | Income (GBP) (ref. 18,000 to 30,999) | | *Less than 18,000* | | | | | |  |  | 1.04 [0.94-1.15] | 4.35x10^-1^ | 1.04 [0.95-1.15] | 4.00x10^-1^ | 1.04 [0.94-1.15] | 4.21x10^-1^ | 1.04 [0.94-1.15] | 4.80x10^-1^ |
|  |  | *31,000 to 51,999* | | | | | | |  |  | 1.00 [0.91-1.10] | 9.63x10^-1^ | 1.00 [0.91-1.09] | 9.40x10^-1^ | 1.00 [0.91-1.09] | 9.33x10^-1^ | 1.01 [0.92-1.11] | 8.62x10^-1^ |
|  |  | *Greater than 52,000* | | | | | | |  |  | 0.94 [0.85-1.04] | 2.64x10^-1^ | 0.94 [0.85-1.04] | 2.63x10^-1^ | 0.95 [0.85-1.05] | 2.83x10^-1^ | 0.96 [0.87-1.07] | 4.60x10^-1^ |
|  | Occupation (ref. Employed (Other)) | | | *Healthcare worker* | | | | |  |  | 1.08 [0.99-1.18] | 6.74x10^-2^ | 1.08 [1.00-1.18] | 5.87x10^-2^ | 1.08 [1.00-1.18] | 6.04x10^-2^ | 1.08 [0.99-1.18] | 7.29x10^-2^ |
|  |  | | | *Unemployed* | | | | |  |  | 1.01 [0.93-1.10] | 8.40x10^-1^ | 1.01 [0.92-1.10] | 8.77x10^-1^ | 1.02 [0.93-1.11] | 6.96x10^-1^ | 1.02 [0.93-1.11] | 6.75x10^-1^ |
|  |  | | | *Retired* | | | | |  |  | 1.02 [0.91-1.14] | 7.52x10^-1^ | 1.01 [0.91-1.13] | 8.30x10^-1^ | 1.02 [0.91-1.14] | 6.92x10^-1^ | 1.03 [0.92-1.16] | 5.51x10^-1^ |
| HRF | Smoking status (ref. Never) | | | | | | *Former* | |  |  |  |  | 1.03 [0.95-1.11] | 5.17x10^-1^ | 1.03 [0.95-1.12] | 4.74x10^-1^ | 1.03 [0.95-1.12] | 4.99x10^-1^ |
|  |  | | | | | | *Current* | |  |  |  |  | 0.94 [0.86-1.02] | 1.14x10^-1^ | 0.94 [0.87-1.02] | 1.43x10^-1^ | 0.94 [0.86-1.02] | 1.36x10^-1^ |
|  | Alcohol drinker status (ref. Current) | | | | | | *Former* | |  |  |  |  | 1.03 [0.96-1.11] | 4.25x10^-1^ | 1.03 [0.96-1.11] | 3.79x10^-1^ | 1.04 [0.96-1.12] | 3.63x10^-1^ |
|  |  | | | | | | *Never* | |  |  |  |  | 0.98 [0.91-1.07] | 7.08x10^-1^ | 0.99 [0.91-1.07] | 7.60x10^-1^ | 0.99 [0.91-1.07] | 7.36x10^-1^ |
|  | BMI (kg/m^2^) (ref. <25) | | | | | | *[25.30[* | |  |  |  |  | 1.08 [0.99-1.19] | 9.90x10^-2^ | 1.09 [0.99-1.19] | 8.20x10^-2^ | 1.09 [0.99-1.20] | 7.49x10^-2^ |
|  |  | | | | | | *[30,40[* | |  |  |  |  | 1.08 [0.98-1.18] | 1.13x10^-1^ | 1.08 [0.98-1.19] | 1.14x10^-1^ | 1.09 [0.99-1.19] | 9.38x10^-2^ |
|  |  | | | | | | *>=40* | |  |  |  |  | 1.03 [0.95-1.11] | 4.55x10^-1^ | 1.03 [0.95-1.12] | 4.39x10^-1^ | 1.03 [0.95-1.12] | 4.08x10^-1^ |
| M | Cancer (ref. No) | | | | | | *Yes* | |  |  |  |  |  |  | 0.93 [0.86-1.00] | 6.59x10^-2^ | 0.93 [0.85-1.00] | 5.96x10^-2^ |
|  | Cardiovascular (ref. No) | | | | | | *Yes* | |  |  |  |  |  |  | 0.94 [0.86-1.02] | 1.41x10^-1^ | 0.94 [0.86-1.02] | 1.53x10^-1^ |
|  | Hypertension (ref. No) | | | | | | *Yes* | |  |  |  |  |  |  | 1.02 [0.93-1.12] | 6.79x10^-1^ | 1.02 [0.93-1.12] | 6.57x10^-1^ |
|  | Diabetes (ref. No) | | | | | | *Yes* | |  |  |  |  |  |  | 1.06 [0.98-1.15] | 1.52x10^-1^ | 1.06 [0.97-1.15] | 1.80x10^-1^ |
|  | Respiratory (ref. No) | | | | *Yes* | | | |  |  |  |  |  |  | 1.01 [0.93-1.09] | 8.16x10^-1^ | 1.01 [0.93-1.09] | 8.57x10^-1^ |
|  | Autoimmune (ref. No) | | | | *Yes* | | | |  |  |  |  |  |  | 0.95 [0.88-1.03] | 2.30x10^-1^ | 0.95 [0.88-1.03] | 2.42x10^-1^ |
|  | Number of medications (ref. 0) | | | | *1* | | | |  |  |  |  |  |  | 1.03 [0.95-1.13] | 4.52x10^-1^ | 1.03 [0.95-1.13] | 4.50x10^-1^ |
|  |  | | | | | | | *>1* |  |  |  |  |  |  | 0.97 [0.88-1.07] | 5.46x10^-1^ | 0.97 [0.87-1.07] | 5.18x10^-1^ |
| E | NO_X_ (ug/m^3^) | | | | | |  | |  |  |  |  |  |  |  |  | 1.05 [0.91-1.21] | 4.89x10^-1^ |
|  | PM10 (ug/m^3^) | | | | | |  | |  |  |  |  |  |  |  |  | 0.96 [0.87-1.06] | 4.65x10^-1^ |
|  | PM2.5 (absorbance/m) | | | | | |  | |  |  |  |  |  |  |  |  | 0.95 [0.85-1.06] | 3.68x10^-1^ |
|  | PM2.5 (ug/m^3^) | | | | | |  | |  |  |  |  |  |  |  |  | 1.16 [1.00-1.33] | 4.24x10^-2^ |
